# A Lassa virus mRNA vaccine confers protection but does not require neutralizing antibody in a guinea pig model of infection

**DOI:** 10.1038/s41467-023-41376-6

**Published:** 2023-09-12

**Authors:** Adam J. Ronk, Nicole M. Lloyd, Min Zhang, Caroline Atyeo, Hailee R. Perrett, Chad E. Mire, Kathryn M. Hastie, Rogier W. Sanders, Philip J. M. Brouwer, Erica Olmann Saphire, Andrew B. Ward, Thomas G. Ksiazek, Juan Carlos Alvarez Moreno, Harshwardhan M. Thaker, Galit Alter, Sunny Himansu, Andrea Carfi, Alexander Bukreyev

**Affiliations:** 1 Department of Pathology, University of Texas Medical Branch, Galveston, TX 77555 US; 2 Galveston National Laboratory, University of Texas Medical Branch, Galveston, TX 77555 US; 3 Department of Immunology and Infectious Diseases, Harvard T.H. Chan School of Public Health, Cambridge, MA 02139 US; 4 Department of Integrative Structural and Computational Biology California Campus, Scripps Research, La Jolla, CA 92037 US; 5 Department of Microbiology & Immunology, University of Texas Medical Branch, Galveston, TX 77555 US; 6 Center for Infectious Disease and Vaccine Research, La Jolla Institute for Immunology, La Jolla, CA 92037 US; 7 Department of Medical Microbiology, Academic Medical Center, Amsterdam, Netherlands; 8 Moderna, Inc, Cambridge, MA 02139 US

**Keywords:** RNA vaccines, Arenaviruses, Viral infection

## Abstract

Lassa virus is a member of the *Arenaviridae* family, which causes human infections ranging from asymptomatic to severe hemorrhagic disease with a high case fatality rate. We have designed and generated lipid nanoparticle encapsulated, modified mRNA vaccines that encode for the wild-type Lassa virus strain Josiah glycoprotein complex or the prefusion stabilized conformation of the Lassa virus glycoprotein complex. Hartley guinea pigs were vaccinated with two 10 µg doses, 28 days apart, of either construct. Vaccination induced strong binding antibody responses, specific to the prefusion conformation of glycoprotein complex, which were significantly higher in the prefusion stabilized glycoprotein complex construct group and displayed strong Fc-mediated effects. However, Lassa virus-neutralizing antibody activity was detected in some but not all animals. Following the challenge with a lethal dose of the Lassa virus, all vaccinated animals were protected from death and severe disease. Although the definitive mechanism of protection is still unknown, and assessment of the cell-mediated immune response was not investigated in this study, these data demonstrate the promise of mRNA as a vaccine platform against the Lassa virus and that protection against Lassa virus can be achieved in the absence of virus-neutralizing antibodies.

## Abstract (web summary)

Lassa virus infections in humans can result in severe disease, including hemorrhagic fever. Here the authors describe an mRNA-based Lassa virus vaccine that shows protection without requirement for neutralizing antibody in a guinea pig model of infection.

## Introduction

Lassa virus (LASV) is the causative agent of Lassa fever, with approximately 100,000–300,000 human cases each year resulting in approximately 5000 deaths^1^. Human infections with LASV were first described in 1969 in Lassa, Nigeria, after two missionary nurses were infected and subsequently succumbed^2^. An Old-World arenavirus, LASV is an enveloped, negative-stranded RNA virus that utilizes an ambisense coding strategy^1^. The LASV genome consists of two segments: the large (L) segment that codes for the viral RNA-dependent RNA polymerase and the Z matrix protein and the small (S) segment that codes for the NP and GPC proteins^1^. LASV GPC is the primary target for virus-neutralizing antibodies since it is the only membrane-anchored protein on the viral envelope^3^. On the surface of the virion, prior to fusion with the host cell, GPC exists as a trimer of heterotrimers^4^. The GPC is cleaved by SKI-1/S1P proteases into GP1, responsible for receptor binding, and GP2, responsible for viral fusion with the host-cell membrane^4^. The GPC protein can exist in two alternative conformations: prefusion and postfusion. The prefusion structure was recently elucidated after 10 years of research^4^. Through the study of hundreds of antibodies from LASV survivors, it was found that most neutralizing antibodies bind to quaternary epitopes on prefusion GPC and require an association of GP1 and GP2^4^. Some of these antibodies have demonstrated cross-neutralization and cross-protection against lethal challenge by multiple LASV clades in guinea pigs^3,5^.

LASV infection is typically asymptomatic or presents with mild, non-specific symptoms in around 80% of those infected, so many cases go unreported^6^. The onset of severe disease is typically characterized by respiratory distress, facial swelling, and hemorrhage^1^. Less common symptoms of severe disease include shock, coma, and seizures^1^. Fifteen to twenty percent of those who are hospitalized succumb within 2 weeks of symptom onset^1^. The most common pathological features seen in LASV infection include focal cytoplasmic degeneration of hepatocytes, multifocal hepatocellular necrosis, monocytic reaction to necrotic hepatocytes, and hepatocellular mitoses^7^. Moreover, in those who recover from the disease, there is a chance of developing temporary or permanent sensorineural hearing loss in one or both ears^6^.

LASV is most common in areas where public health measures are difficult to implement, access to healthcare is limited, and contact with peridomestic rodents and their secretions still occurs^1,8^. Normally, LASV is transmitted to humans via contact with its rodent reservoir, *Mastomys natalensis* (Natal multimammate mouse)^6,8^. These rodents live in and around houses, and the virus is usually inhaled via aerosolized rodent excretion and secretions from cleaning or consumption of contaminated food. In recent years, LASV has been isolated from several additional rodent species, *Mastomys erythroleucis*, *Hylomyscus pamfi* and *Mus baoulei*^9,10^. However, human-to-human transmission has been documented, and LASV has also been seen to persist in human urine and semen months post-exposure^1,11–13^. Human-to-human transmission of LASV is particularly concerning because the diagnosis is confounded by the presence of more common endemic diseases showing similar symptoms^1^. Additionally, LASV has been repeatedly exported outside of endemic regions. In February 2022 in the United Kingdom, there were three cases, including a fatal one, in a family returning from vacation in Mali^14^. Additionally, there have been exported cases documented in Japan, Israel, Germany, Canada, the Netherlands, and the United States^15,16^.

LASV is extremely heterogeneous and has four well-known lineages or clades, and additional lineages were discovered in the last decade^17^. LASV has been added to the World Health Organization R&D blueprint list of diseases that urgently require accelerated research and is one of four diseases targeted by the Coalition for Epidemic Preparedness Innovations (CEPI) for vaccine development^18–20^. Despite the significant burden of disease, there have been no specific treatments or vaccines approved, and in recent years, outbreaks have been increasing in severity^21–23^.

The recently developed modified-mRNA vaccine platform has multiple advantages: it is highly immunogenic, non-infectious, lacks viral vector or another carrier that could induce non-desirable immune responses, and lacks a risk of incorporation into the host’s genome^24^. The immunogenicity of vaccines based on conventional mRNA may be reduced due to induction of the innate immune response^25^ that can lead to suppression of translation^26^. In addition, induction of the innate immune response results in the degradation of cellular and ribosomal RNA^26^. However, several nucleoside modifications have been designed to combat induction of the innate immune response, of which replacement of uridine with pseudouridine or N1-methylpseudouridine has been proven to be the most optimal^26,27^. RNA that includes pseudouridine is not recognized by toll-like receptors, resulting in reduced stimulation of the innate immune response, increased translation, and improved immunogenicity^27,28^. In addition, pseudouridine is a safe and naturally occurring modification of mRNA^24,28^. mRNA vaccine constructs are packaged in lipid nanoparticles (LNPs), which serve two purposes: delivery of the mRNA in the cytoplasm of cells and its protection from nucleases^24^. The LNP-formulated modified N1-methylpseudouridine mRNA platform has been used in the COVID-19 BioNTech & Pfizer and Moderna vaccines which received emergency use authorization from the FDA and EMA and are now fully approved for those over 5 years of age^29^.

Here, we have developed and tested an N1-methylpseudouridine-modified RNA vaccine encapsulated in lipid nanoparticles that encodes either wild-type (WT) LASV GPC or the prefusion stabilized GPC. Our data demonstrates the induction of non-neutralizing antibody responses, fc-mediated antibody effector mechanisms, and complete protection against lethal LASV challenge by a mRNA vaccine in the guinea pig model.

## Results

### Design and generation of the vaccine constructs

**Fig. 1:**
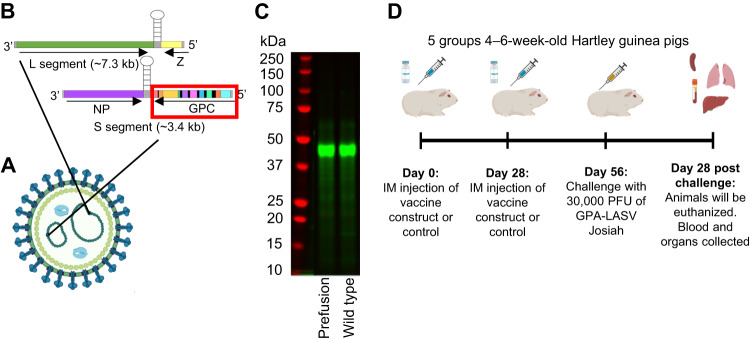
Generation of mRNA-based vaccine. **A** The structure of LASV particle. **B** LASV L (green and yellow) and S (purple and multi-color for the GPC) segments. **C** SDS PAGE gel image of in vitro transcribed prefusion and wild-type LASC GPC vaccine constructs. This was run once. **D** Animal study outline: schedule for two doses of vaccine, challenge, and end of study. Each group included 5 animals, for a total of 25 animals in the study. The animals were 4–6 weeks old and had a starting weight of 350–400 g. The control group received two doses of sterile phosphate-buffered saline. Graphics were generated by N. Lloyd with Biorender.com and Microsoft PowerPoint.

We designed mRNA encoding the WT Lassa clade IV strain Josiah GPC and its prefusion-stabilized version by the introduction of a series of mutations: E329P in the metastable region of HR1 in GP2, cysteine linkages of GP1 and GP2 at R207C and G360C, and the replacement of the native S1P GP1-GP2 cleavage site with a furin site (RRLL to RRRR)^4^. Linearized DNA templates were used for in vitro synthesis of modified mRNA by T7 polymerase-mediated transcription in which the UTP was substituted with 1-methylpseudo-UTP. mRNA constructs were then packaged in lipid nanoparticles before being used in immunization experiments (Fig. 1A–C).

### Vaccination with either WT or prefusion-stabilized constructs elicited robust binding antibody titers to the prefusion-stabilized GPC but not WT GPC

**Fig. 2:**
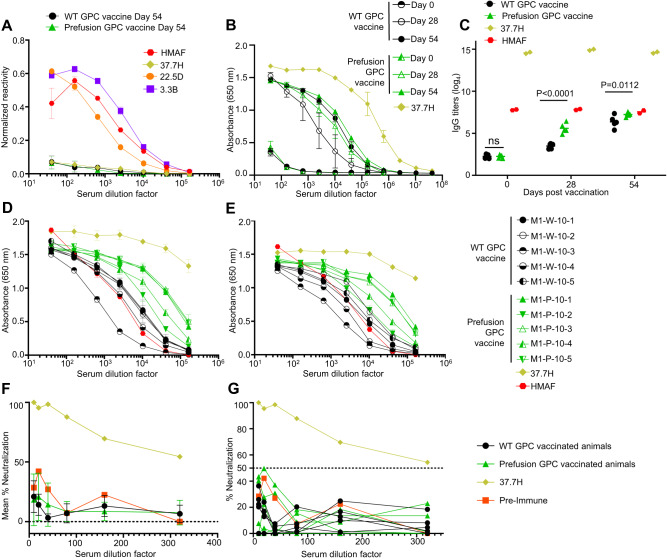
Antibody response to vaccination with WT of prefusion-stabilized GPC mRNA. **A** Quantification of IgG binding to postfusion LASV GPC determined by ELISA. Controls: HMAF from LASV-infected mice, 37.7H prefusion GPC-specific mAb, 22.5D GP2 specific mAb, and 3.3B a GP1 specific mAb. Pooled serum from 10 guinea pigs per vaccine. **B** Quantification of IgG binding to prefusion-stabilized LASV GPC determined by ELISA. Each symbol represents the average values of one group. *N* = 5. For (**A**) and (**B**), the error bars represent the standard deviation of the means of the 5 animals per group. **C** ELISA binding antibody titers, endpoint dilution, with prefusion GPC antigen from panel (**B**) (Two-way ANOVA), ns *P*-value = 0.998. Controls: HMAF and 37.7H. **D** Binding of IgG to prefusion stabilized Clade 2 (strain NIGA08-41) LASV GPC determined by ELISA. Ten animals shown individually. **E** Binding of IgG to prefusion stabilized Clade 5 (strain Soromba-R) LASV GPC determined by ELISA. Ten animals shown individually. For (**D**) and (**E**), the error bars represent the standard deviation between the duplicate values run. **F** Mean neutralization curves for both vaccine groups on day 54. *N* = 5. The error bars represent the standard deviation of the means of the 5 animals per group. **G** Individual neutralization curves for each animal on day 54—*N* = 5 per vaccine or control group. For all assays in this figure, samples were run in duplicates, and each assay was performed at least twice. Throughout, WT GPC vaccinated animals are represented by a filled-in black circle. Prefusion GPC vaccinated animals are represented by a filled-in green triangle. Antibody control, 37.7H is represented by a mustard-colored diamond. Serum control, HMAF, is represented by a red hexagon. Pre-immune serum control is represented by a filled-in orange square. Source data are provided as a Source Data file.

Six- to eight-week-old, outbred Hartley guinea pigs were vaccinated intramuscularly 28 days apart with 10 µg of either the LNP-formulated WT or prefusion stabilized GPC constructs, and the control animals were inoculated with phosphate-buffered saline (PBS) (Fig. 1D). Serum samples were collected immediately prior to each vaccination and at day 54—immediately prior to transfer to ABSL-4 containment. The vaccine was well tolerated, with no signs of clinical distress or weight loss and no signs of inflammation at the sites of injection (Supplementary Fig. 1A, B). The binding of antibodies to LASV GPC was analyzed by enzyme-linked immunosorbent assays (ELISA) using two alternative antigens. The first antigen—a crude lysate of irradiated LASV strain Josiah-infected Vero E6 cells—allows the detection of antibodies that bind the WT, postfusion, form of the LASV GPC. Indeed, we found that hyperimmune mouse ascites fluid (HMAF) from LASV-infected mice was able to bind the lysate, whereas no binding was detected when we used 37.7H, a known neutralizing prefusion-GPC-specific LASV antibody^4^ (Fig. 2A). Interestingly, animals vaccinated with neither WT nor prefusion stabilized GPC developed significant antibody responses against the lysate (Fig. 2A). To specifically detect antibodies binding GPC in the prefusion conformation, we used a second antigen that consisted of a prefusion-stabilized LASV strain Josiah GPC fused to the I53-50A.1NT1 scaffold^4,30^. Fusion of prefusion GPC to I53-50A.1NT1 was previously shown to stabilize the trimeric conformation of GPC^30^. Vaccination with either construct elicited antibody responses with a strong reactivity to GPC-I53-50A.1NT1, with the prefusion stabilized GPC vaccinated animals developing significantly higher titers after the second dose and after the challenge (Fig. 2B, C). In addition to high titers against the LASV clade IV prefusion-stabilized recombinant protein, animals vaccinated with either construct also elicited robust binding antibody responses to previously described prefusion-stabilized GPC-I53-50A.1NT1 proteins of the LASV clade II (strain NIG08-A41) and LASV clade V (strain Soromba-R) genotypes (Fig. 2D, E)^31^. Animals vaccinated with the prefusion stabilized GPC-based vaccine produced markedly higher titers against both LASV clades II and V compared to the wild-type GPC-based vaccinated animals (Fig. 2D, E).

### Vaccination with either the WT or prefusion-stabilized construct elicited LASV-neutralizing antibody response in some, but not all animals

Neutralizing antibody response against LASV strain Josiah was determined in serum samples from vaccinated animals collected two days prior to the challenge and pre-immune samples. As a positive control, we used 37.7H, which demonstrated 100% neutralization (Fig. 2F, G) and IC50 of 6.92 µg/mL^3^. In the WT GPC mRNA group, none of the sera samples demonstrated neutralizing activity over 40%, which was comparable to the neutralizing activity seen in the pre-immune serum. In the prefusion stabilized GPC vaccinated group, two out of five animals demonstrated neutralizing activity above pre-immune values at nearly 50% (Fig. 2F, G). The prefusion GPC-vaccinated group had greater levels of virus-neutralizing activity compared to the WT GPC-vaccinated group, although the difference between the two groups and the pre-immune serum activity was not significant. The observed animal-to-animal variation in neutralizing antibody activity is consistent with the outbred animal model used.

### Vaccination with either the WT or prefusion stabilized construct primarily elicited antibodies specific to linear epitopes in the N-terminal domain of GP1 and T-loop of GP2

**Fig. 3:**
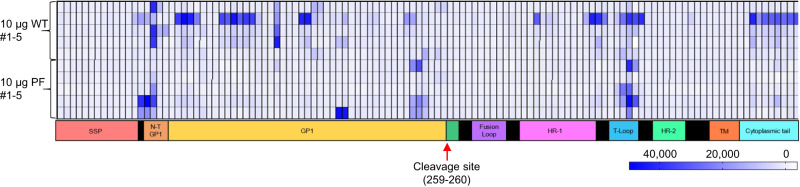
Peptide array mapping of linear epitopes associated with the vaccine-induced humoral response in vaccinated animals. Array comprised 120 peptides spanning the length of the 491 aa LASV GPC. Each 15-mer peptide overlaps the next by 11 amino acids. Stable signal peptide (SSP) in peach, N-terminal domain (NTD) of GP1 in tangerine, GP1 in yellow, fusion loop in violet, HR-1 in pink, T-loop in light blue, HR-2 in mint green, TM in orange, cytoplasmic tail in cyan. Each sample was run in triplicate. Source data are provided as a Source Data file.

To test antibody response against linear epitopes, we used an array of 120 15-mer peptides overlapping by 11 amino acids, which span the entire GPC of LASV strain Josiah. In animals vaccinated with the WT GPC construct, linear epitopes were largely identified in the N-terminal domain (NTD) of GP1 with some scattered binding across GP1 and binding to the T-loop in GP2 (Fig. 3). This pattern was consistent with all animals in this group except for animal 2 in the WT GPC vaccinated group, which displayed binding to additional epitopes across GP1 and the cytoplasmic tail. This same animal demonstrated the greatest level of neutralizing antibody response of all vaccinated animals, either WT or prefusion (Fig. 2F, G). In animals vaccinated with the prefusion-stabilized construct, linear epitopes were similarly identified in the NTD region of GP1 and in the T-loop of GP2 (Fig. 3). These two regions are known to interact in the prefusion structure of GPC^4^. Additionally, these are the regions at the base of the prefusion trimer where 37.7H is known to bind^4^. However, animals that received the prefusion-stabilized construct elicited antibodies that were generally more focused on specific domains compared to WT, where additional sporadic linear epitopes were identified. Thus, both vaccine constructs induced antibodies mostly, but not exclusively, specific for linear epitopes in NTD and T-loop.

### Vaccination with either the WT or the prefusion stabilized construct induces polyclonal antibody responses directed at known prefusion GPC epitopes

**Fig. 4:**
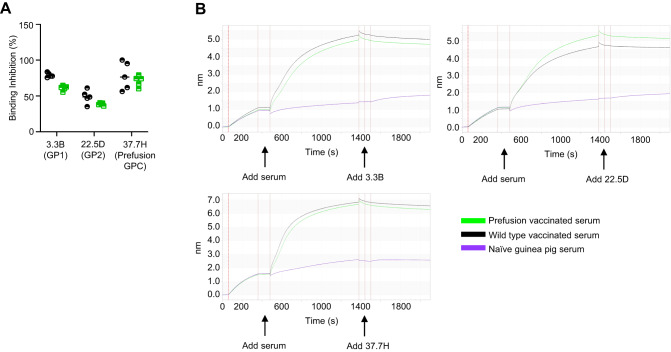
Mapping of the antibody response by biolayer interferometry competition binding assay with mAbs of known epitope specificity. **A** Inhibition of binding of the immune sera by the indicated mAbs. The antigen (GPmperP) is the non-prefusion stabilized, uncleaved GPC that contains a linker between GP1 and GP2. This protein adopts both the pre-fusion and the post-fusion forms. Values normalized to 100%. **B** Representative data curves for mAbs 37.7H, 3.3B, and 22.5D with vaccinated and naïve guinea pig sera. Samples were run in duplicate, and the assay was performed twice. *N* = 5 animals per study group. Source data are provided as a Source Data file.

In an attempt to further map the antibody response, we tested the ability of the immune sera to compete with well-characterized GPC-specific human mAbs for binding to GPC by biolayer interferometry. The following mAbs were used: 3.3B, 22.5D, and 37.7H^3,4^. 37.7H is known to specifically bind to a bipartite site (two adjacent monomers) at the base of the prefusion trimer that spans across four regions of the LASV GPC^4^. These sites include the NTD and HR1 of GP1 and the T-loop and HR1 of GP2^4^. 3.3B and 22.5D are not neutralizing but are specific for the LASV GP1 and GP2, respectively^3^. Highly neutralizing antibodies against LASV are known to bind the quaternary prefusion assembly of the LASV GPC rather than any single GPC subunit^4^. These antibodies are thought to interfere with the viral cell entry by preventing virus binding to the cell receptor α-dystroglycan or fusion of the viral and host-cell membranes^32^. Streptavidin sensors were coated with GPmperP, a flexible version of the LASV Josiah GPC that adopts both the pre- and post-fusion conformations^4^. Coated sensors were first incubated with immune sera, then one of the three mAbs, and the magnitude of binding was measured (Fig. 4A). All three mAbs appeared to compete with the immune sera from either vaccine group. The highest binding inhibition percentage on average was seen with 37.7H, consistent with the IgG ELISA data (Fig. 2B, C), followed by 3.3B, and lastly 22.5D (Fig. 4A). These data demonstrate that (1) the antibody responses are directed against both GP1 and GP2; (2) the vaccines include antibodies specific to not only linear but also quaternary epitopes; and (3) the two vaccines induce antibody responses with similar antigenic specificity (Fig. 4A, B).

### Vaccination with either the WT or the prefusion stabilized construct significantly stimulates Fc-mediated antibody effector mechanisms

**Fig. 5:**
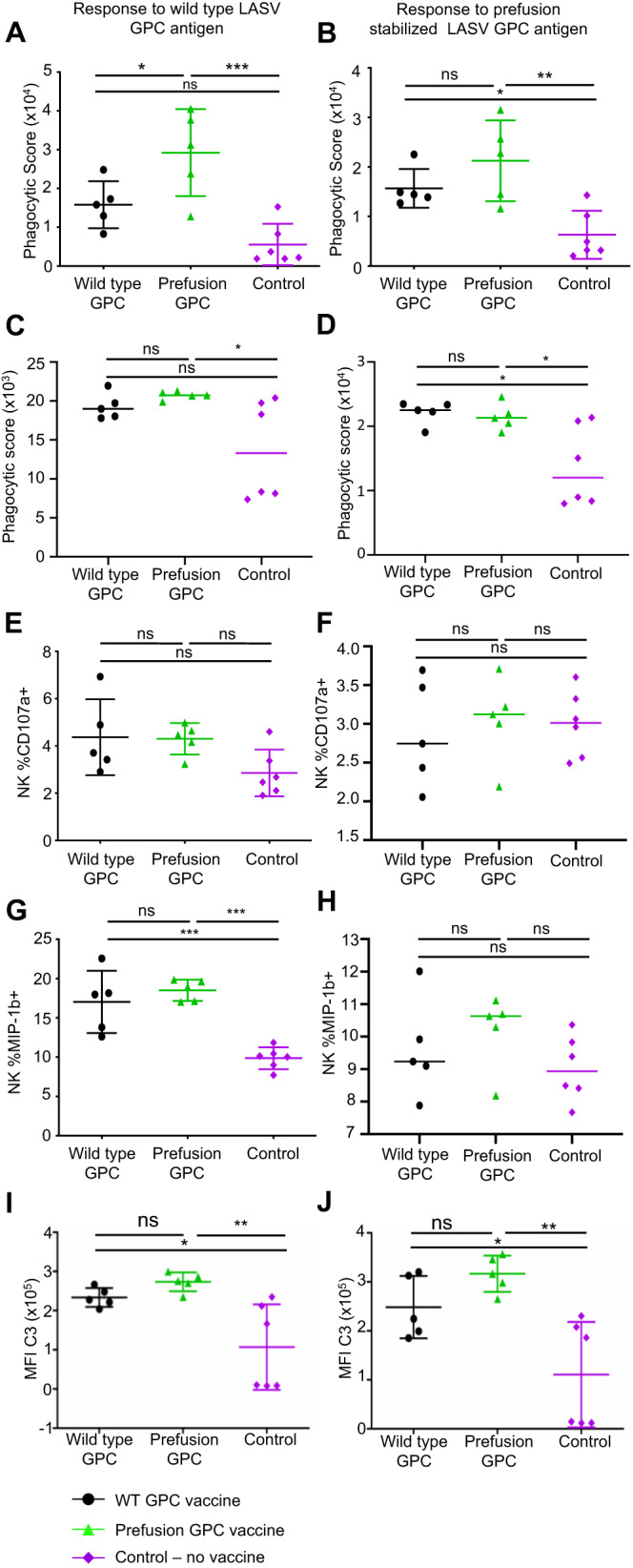
Fc-mediated effector functions of the immune sera after vaccination with wild type or prefusion-stabilized mRNA constructs. **A**, **B** Antibody-dependent neutrophil phagocytosis (ADNP). **C**, **D** Antibody-dependent cellular phagocytosis (ADCP). **E**, **F** Percentages of NK cells positive for CD107a. **G**, **H** Percentages of NK cells positive for MIP-1$\beta$. **I**, **J** Antibody-dependent complement deposition (ADCD). Antibody response to wild-type LASV GPC vaccine (**A**, **C**, **E**, **G**, **I**) and prefusion stabilized LASV GPC vaccine (**B**, **D**, **F**, **H**, **J**). Ns, not significant; * *P* < 0.05; ** *P* < 0.01; *** *P* < 0.001. One-way ANOVA with Tukey’s post-hoc test. **A** ns, *P* = 0.1145. **B** ns, *P* = 0.3029. **C** Between WT and PreF ns, *P* = 0.8455, between WT and control ns, *P* = 0.0955. **D** ns, *P* = 0.966. **E** Between WT and PreF ns, *P* = 0.9949. Between WT and control ns, *P* = 0.1120. Between PreF and control ns, *P* = 0.1326. **F** Between WT and PreF ns, *P* = 0.8805. Between WT and control ns, *P* = 0.9325. Between PreF and control ns, *P* = 0.9881. **G** ns, *P* = 0.6163. **H** Between WT and PreF ns, *P* = 0.7593. Between WT and control ns, *P* = 0.7045. Between PreF and control ns, *P* = 0.2990. **I** ns, *P* = 0.6545. **J** ns, *P* = 0.3827. For (**A**, **B**, **E**, **F**, **G**, and **H**), assays were run with PBMCs isolated from two separate donors. On all charts, the mean of the 5 animals per group and the standard deviation of the 5 animals per group is represented with error bars. For all figures, WT GPC vaccinated animals represented by a filled-in black circle. Prefusion GPC vaccinated animals represented by a filled-in green triangle. All non-vaccinated control animals represented but a filled-in purple diamond. For all figures, *N* = 5 animals per group. Source data are provided as a Source Data file.

As LASV-neutralizing antibody responses were not induced in all animals, despite the excellent protection observed in each vaccinated animal, we investigated alternative mechanisms of antibody-mediated protection. The mechanisms tested were antibody-dependent neutrophil phagocytosis (ADNP), antibody-dependent cellular phagocytosis mediated by monocytes (ADCP), antibody-dependent NK cell activation (ADNKA), and antibody-dependent complement deposition (ADCD). Each of the mechanisms was tested in response to the WT and prefusion-stabilized LASV antigen (Fig. 5). ADNP was induced in the two vaccinated groups and was significantly greater in the prefusion-stabilized vaccinated group compared to WT in response to only the WT GPC (Fig. 5A, B). ADCP was significantly induced for the prefusion vaccinated animals when measured with both the WT and the prefusion antigens, whereas vaccination with the WT construct only significantly increased ADCP when measured in response to the prefusion antigen (Fig. 5C, D). For analysis of ADNKA, we measured percentages of NK cells positive for CD107a, a marker for degranulation, and MIP-1*β*, a marker of NK cell activation. Neither vaccine construct significantly increased the percentage of CD107a^+^ NK cells compared to the control (Fig. 5E, F). However, the percentage of MIP-1*β*^+^ NK cells was significantly increased in both vaccine groups in response to the WT antigen only (Fig. 5G, H). ADCD was markedly greater for sera from both WT GPC and prefusion-stabilized GPC-vaccinated animals compared to the control animals, although some of the differences were not significant due to high animal-to-animal variation (Fig. 5I, J). Overall, vaccination with either construct induced each of these Fc-mediated immune mechanisms.

### Both the WT and the prefusion-stabilized constructs confer protection against death and severe disease caused by lethal dose of LASV

**Fig. 6:**
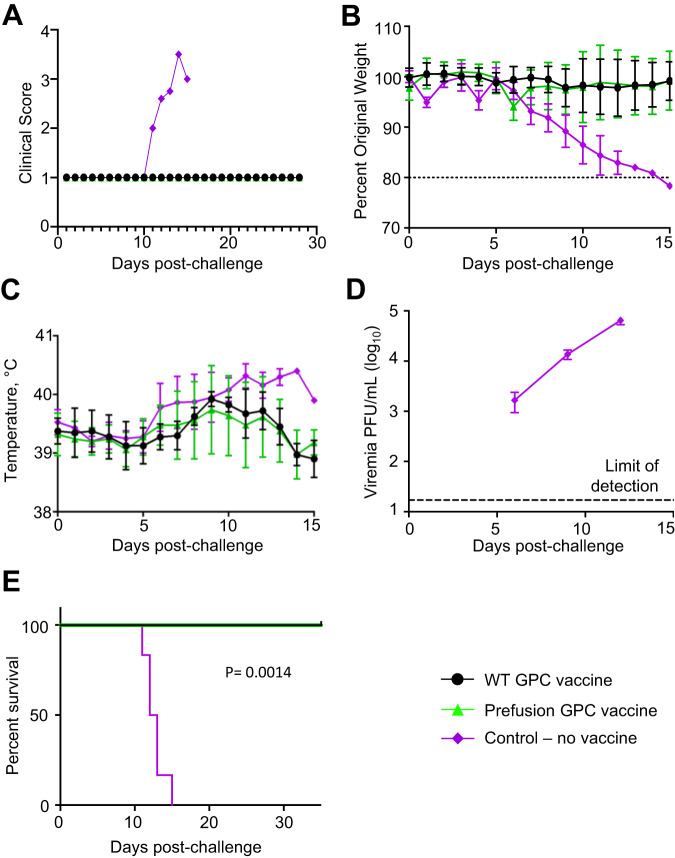
Protective efficacy in vaccinated guinea pigs. **A** Disease score. **B** Weight change. Dashed line represents 20% weight loss (animal at 80% of the original weight) which was the weight cutoff for euthanasia. **C** Body temperature, **D** Viremia. Lower limit of detection is 50 PFU/mL. Only the control group is included, as no viremia was detected in either vaccinated groups. For (**B**–**D**), error bars represent the standard deviation between the averages of the 5 animals per group. **E** Percent survival. Mean values based on 5 animals per group ± SD. *P* = 0.0014 for both WT GPC vaccine versus control and prefusion GPC vaccine versus control. Log-rank (Mantel-Cox) test. For all figures, WT GPC vaccinated animals represented by a filled-in black circle. Prefusion GPC vaccinated animals represented by a filled-in green triangle. All non-vaccinated control animals represented but a filled-in purple diamond. For all figures, each group is an average of the *N* = 5 animals per group. Source data are provided as a Source Data file.

On study day 56, the animals were challenged intraperitoneally with 30,000 PFU of guinea pig-adapted LASV strain Josiah^33^ (Fig. 1D). Weights, temperature, and disease scores were recorded at least daily. Control animals did not display lethargy, ruffled fur, weight loss, or other signs of disease until 7 to 9 days post-challenge, at which point they became febrile (Fig. 6A). Fever persisted until animals became moribund, at which point body temperature tended to drop sharply. Animals began to lose weight by day 8 post-challenge and began to show clinical signs of disease, notably lethargy, ruffled fur, and orbital tightening (Fig. 6B, C, Supplementary Fig. 2). All control animals became viremic by day 9, developed the disease, met criteria for euthanasia (defined in “Methods”), and were euthanized on days 11–15 (Fig. 6D, E). In contrast, all vaccinated animals in both WT GPC and prefusion GPC-stabilized groups showed no outward signs of disease (as defined in “Methods”), and all survived the challenge to day 28 when animals were euthanized (Fig. 6E, Supplementary Fig. 2), except up to 0.65 ± 0.37 °C mean transient increase in the temperature (not significant due to variation between individual animals) (Fig. 6C).

Analysis of sera from the terminal bleeding demonstrated a marked increase in postfusion GPC-specific antibodies but no significant increase in prefusion GPC-specific antibodies (Supplementary Fig. 3A). Additionally, antibodies against LASV NP protein were detected (Supplementary Fig. 3B, C). These NP antibodies are likely to be a result of low-level LASV replication despite the lack of detectable virus in serum throughout post-challenge time points.

**Fig. 7:**
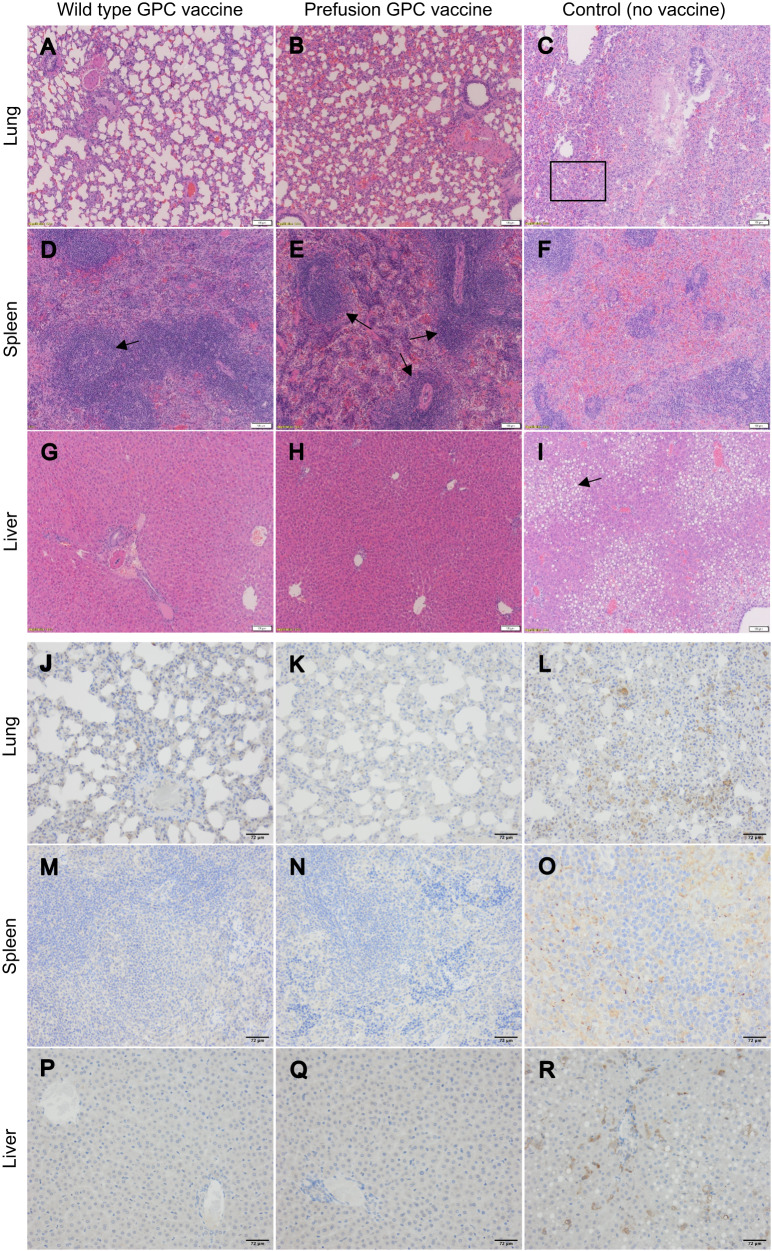
Histopathology and LASV immunohistochemistry in organs of vaccinated and control animals. Images of H&E stained (**A**–**I**) and LASV nucleoprotein immunostained (**J**–**R**) tissues of guinea pig lung (**A**–**C** and **J**–**L**), spleen (**D**, **E** and **M**–**O**) and liver (**G**–**I** and **P**–**R**). Scale bars in bottom right corners of H&E images = 103 μM. IHC images magnified at 20x, scale bars added in ImageJ in bottom right corners = 72 μM. **C** The box indicates a representative area of viral interstitial pneumonia. **D**, **E** The arrows indicate germinal center activation, which is notably more robust in vaccinated animals compared to the unvaccinated control. **I** The arrow shows an area demonstrating steatosis in the liver of the control animal, which is not present in the vaccinated animals. Staining was performed once on duplicate slices of tissue.

Lungs, spleens, and livers were collected from one animal per group (Fig. 7A–I). Additional Animal Studies 1 and 2, in which guinea pigs were vaccinated and challenged to obtain additional tissues for histological examination, were performed (Supplementary Figs. 4–14). Additional Study 1 was performed identically to the original study, while in Additional Study 2, animals were vaccinated and challenged identically to the original study but were euthanized on day 9, which is the expected peak of viral replication^34^ to characterize pathology and presence of the viral antigen by immunohistochemistry (Supplementary Figs. 9–14). In the control group, lungs presented with typical viral interstitial pneumonia^35^ (Fig. 7C and Supplementary Fig. 5). Sinus histiocytosis was the most prominent finding in the spleen (Fig. 7F and Supplementary Figs. 6 and 7). Sections of the liver from control animals demonstrated pathology typical of LASV infection in guinea pigs and consisted of lymphohistiocytic hepatitis and hepatocellular degeneration^35^ (Fig. 7I and Supplementary Figs. 8 and 9). The animal selected for histopathology also presented with a notable steatosis. Marked steatosis was also noted in 4 out of 5 of the control animals in Additional Study 1 (Supplementary Fig. 8). This is a somewhat common but not universal feature associated with a sudden decline in caloric intake. In contrast, animals vaccinated with either vaccine had normal lung and liver tissues (Fig. 7A, G and Supplementary Fig. 8). The vaccinated animals remained healthy till the end of the study. Furthermore, significant immune activity in the spleens of vaccinated animals was evidenced by numerous and prominent white pulp foci and germinal centers (Fig. 7D and Supplementary Fig. 6). Immunohistochemical analysis of the samples corroborated the histopathology data. LASV NP was detectable in the lungs, spleens, or livers of control but not vaccinated animals (Fig. 7J–R and Supplementary Figs. 10 and 11). In Additional Study 2, at day 9 post-challenge, there was some staining for LASV NP observed in vaccinated animals (Supplementary Figs. 12–14). Examination of the H&E-stained and immunostained tissues demonstrated better protection of the animals by the prefusion stabilized GPC-based vaccine (Supplementary Figs. 5, 12, and 13) compared to the wild-type GPC-based vaccine. Overall, mRNA vaccines encoding for either WT GPC or prefusion-stabilized GPC provided protection against death and severe disease caused by LASV in guinea pigs.

## Discussion

In this study, we designed and generated lipid nanoparticle formulated mRNA vaccines, encoding either the WT or the prefusion-stabilized LASV GPC and tested their immunogenicity and efficacy against lethal LASV challenge in outbred Hartley guinea pigs. Both vaccines were safe and well tolerated. Vaccination with either construct induced robust binding antibody titers, with the prefusion stabilized construct vaccine eliciting significantly higher levels of these antibodies. The antibodies induced by either construct demonstrated binding almost exclusively to the prefusion form of the LASV GPC. Contrary to our expectations, prefusion GPC-specific binding antibody titers were high in both the WT and prefusion GPC vaccinated animals. The antibodies produced in response to vaccination with either construct significantly increased ADNP, ADCP, ADNKA, and ADCD. Of note, LASV-neutralizing antibody responses were detected in only some but not all animals in either group. Despite that, all vaccinated animals were protected from death and severe disease caused by LASV. Because of the variable levels of the induced virus-neutralizing antibodies, we hypothesize that the protection may be associated with the Fc-mediated effects. It is also possible that the protection is conferred by the cell-mediated response. As mRNA vaccines produce antigen in cells targeted by LNPs, these vaccines are expected to induce cell-mediated responses. Indeed, induction of a potent cell-mediated response was detected in humans vaccinated with mRNA vaccines against SARS-CoV-2^36^.

Several LASV vaccine candidates, based on various platforms, have demonstrated protection in animal models with and without demonstrating neutralization. LASV vaccines that have demonstrated production of LASV-neutralizing antibodies include the live-attenuated MOPEVAC_LAS_, two vesicular stomatitis virus (VSV) vectored vaccines, the monovalent VSVΔG/LVGPC and the quadrivalent VesiculoVax vaccine (against Ebola virus, Sudan virus, Marburg virus, and LASV) and the DNA vaccine, pLASV-GPC^37–41^. For MOPEVAC_LAS_, LASV-neutralizing antibodies were detected in vaccinated animals, and for MOPEVAC_LAS_, LASV-specific TNFα^+^ CD4^+^ and TNFα^+^ CD8^+^ T cells were also detected. Both VSVΔG/LVGPC and VesiculoVax induced moderate to high titers of serum IgG along with moderate virus-neutralizing antibody titers to both glycoproteins^42^. For pLASV-GPC in NHPs, complete protection was achieved after two doses of pLASV-GPC DNA four weeks apart^40^. LASV GPC-specific binding antibodies were detected in three out of six macaques after the first vaccine dose and in all six animals after the second dose, and LASV-pseudovirus neutralizing activity was detected in all animals after the second dose^40^.

Vaccines that did not demonstrate any significant virus-neutralizing antibody response despite robust protection and, for some, induction of non-neutralizing antibodies include the measles virus (MeV) vectored constructs, MeV-Z and MeV-NP^43^, and the rhabdovirus-vectored LASV vaccine construct^44^. There were no significant LASV-specific IgG titers in macaques for either the MeV-Z or MeV-NP constructs despite the near-sterilizing protection seen in the MeV-NP group^43^. Only one of 4 of the MeV-NP vaccinated animals had any virus-neutralizing antibody response^43^. For the rhabdovirus-vectored construct, vaccinated animals were protected from infection and lethal disease caused by LASV and rabies viruses, despite insignificant virus-neutralizing antibody titers. Vaccination significantly induced LASV-specific non-neutralizing antibodies, ADCP mediated by macrophages and antibody-dependent cellular cytotoxicity mediated by NK cells^44^. The study also demonstrated that the FcγR-receptor function is critical for the vaccine-induced protection in a surrogate VSV-LASV mouse model^44^.

When LASV was first isolated, a virologist contracted the disease and was saved by the successful use of immune serum from Lily Pinneo, a nurse who survived LASV infection^45^. Despite this early success, the use of immune serum or convalescent plasma was later documented not to have a significant therapeutic effect. However, differences in the efficacy of convalescent plasma are likely associated with differing levels of antibody titers. Due to heavy glycan shielding and the metastable nature of the LASV GPC, the induction of neutralizing antibodies is not typically seen until late or post-infection^46^. Recently, 113 monoclonal antibodies were isolated from 17 LASV pre-exposed individuals that maintained antibody titers months to years post-infection. Within this panel, a number of broadly neutralizing antibodies were found^3^. These broadly neutralizing antibodies largely belong to the GPC-B group of antibodies that bind to a conserved quaternary epitope at the base of the LASV GPC that spans both GP1 and GP2, holding the GPC in its prefusion form^4,46,47^. Administration of a cocktail of these antibodies fully protected both outbred guinea pigs and cynomolgus macaques from lethal LASV infection even when administered up to 8 days post-challenge^46,48^.

We sought to compare the efficacy of the LASV GPC in both its wild-type and prefusion-stabilized form. Similar to what has been seen with respiratory syncytial virus, we hypothesized that the use of the prefusion-stabilized conformation of the antigen would yield higher titers of virus-neutralizing antibodies compared to the WT form and ultimately greater efficacy^49^. The prefusion-stabilized conformation of LASV GPC could facilitate the induction of highly neutralizing antibodies that would inhibit infection by preventing fusion with the cell surface^4^. Interestingly, while there was no significant difference in efficacy between the two mRNA vaccine constructs, non-neutralizing antibodies induced in both groups were specific for the prefusion-stabilized LASV GPC. We also found that the prefusion stabilized GPC-based vaccine-induced higher titers against both LASV Clade II and V compared to the wild-type GPC construct, which may indicate that the prefusion construct may perform better in heterologous challenges in future studies. In addition, both vaccines induced comparable ADNP, ADCP, ADNKA, and ADCD.

Limitations of this study include the lack of guinea pig-specific reagents for assessment of cell-mediated immunity and the limited sample volume that prevented further assessment of the vaccine mechanism of protection. However, the lack of reagents is a common issue associated with the assessment of therapeutics for LASV in guinea pigs^50–52^. Future work for this study includes a more in-depth investigation of the mechanism of protection induced by vaccination with our mRNA constructs. We specifically plan to assess the efficacy of the passive transfer of vaccinated immune serum against lethal challenges and assess the contribution of the cell-mediated immune response via depletion of CD4+ T cells, CD8+ T cells, or both. These studies will be possible as guinea pig-specific reagents for the depletion of T cells have recently been developed^53^. We also plan to assess the durability of the immune response and the protective efficacy of a single-dose regimen, as well as protection against heterologous LASV clades. Another limitation of the study is that the guinea pig model, while it represents an important step in the assessment of a LASV vaccine, does not necessarily predict protection in humans; as such, our next step will be testing the protection in non-human primates. Finally, the number of animals included in the study was limited due to the space and other limitations associated with work under BSL-4 containment.

The mRNA vaccine platform is highly versatile and has been shown to be highly efficacious against SARS-CoV-2^54,55^. This platform is highly flexible, which will be imperative for combatting LASV due to its high heterogeneity. Additional benefits of this platform include rapid manufacture and modification of the construct to adjust to changes in the pathogen. The mRNA-based LASV Josiah mRNA vaccines presented in this study demonstrated 100% protection in guinea pigs against death and severe disease. Further studies are required to understand the mechanism of protection of these vaccines and the contributions of the cell-mediated and humoral immune responses in the protection.

## Methods

All of the following research complies with the ethical regulations laid out by the UTMB Institutional Biosafety Committee and IACUC. Additionally, the use of consented and deidentified human NK cells and PBMCs was approved by the MGH Institutional Review Board.

### Generation of the vaccine constructs

The prefusion-stabilized GPC was generated as described previously, via the introduction of a series of mutations: R207C and G360C to link GP1 and GP2, E329P in HR1 of GP2, replacing the S1P GP1-GP2 cleavage site with a furin site (RRLL to RRRR), and the maintenance of the stabilized signal peptide for stabilization of the GPC primer^4^. The mRNA was synthesized as previously described^56^. Briefly, mRNAs were synthesized in vitro using an optimized T7 RNA polymerase-mediated transcription reaction with complete replacement of uridine by N1-mpseudouridine^57^. The reaction included a DNA template containing the immunogen open-reading frame flanked by 5’ untranslated region (UTR) and 3’ UTR sequences and was terminated by an encoded polyA tail. After transcription, a cap 1 structure was added using the vaccinia capping enzyme and mRNA 2ʹ-*O*-Methyltransferase (New England Biolabs). The mRNA was purified by oligo-dT affinity purification, buffer exchanged by tangential flow filtration into sodium acetate, pH 5.0, sterile filtered, and kept frozen at −20 °C until further use.

The LNP formulations were prepared as previously described^58^. Briefly, mRNAs were encapsulated in a lipid nanoparticle through a modified ethanol-drop nanoprecipitation process. Ionizable, structural, helper, and polyethylene glycol lipids were mixed with mRNA in an acetate buffer, pH 5.0, at a ratio of 2.5:1 (lipid:mRNA). The mixture was then neutralized with Tris-HCl, pH 7.5, and sucrose was added as a cryoprotectant. The final solution was sterile-filtered, and vials were filled with formulated lipid nanoparticle and stored frozen at −20 °C until further use. The pre-clinical vaccine product underwent analytical characterization, which included the determination of particle size and polydispersity, encapsulation, mRNA purity, double-stranded RNA content, osmolality, pH, endotoxin, and bioburden, and the material was deemed acceptable for in vivo study. Additionally, to confirm that the chosen construct yielded the correct proteins, cell-free translation coupled with sodium dodecyl sulfate–polyacrylamide gel via electrophoresis was performed to verify the size of the resulting protein. This process checked both protein expression and size without the need for specific detection antibodies. The expected mass of the constructs, both the prefusion and the WT GPC, were approximately 49 kDa (Fig. 1C).

### Animal work

All research was conducted in female outbred Hartley guinea pigs purchased from Charles River. Caesarian derived in 1969, the guinea pig line was originally provided to Charles River in 1968 from the Medical Research Council, Millhill, England. All animal experimentation was performed in accordance with UTMB’s Institutional Animal Care and Use Committee (IACUC) guidelines and ethical practices. Animal infections and necropsies with the experimental agent were performed by trained personnel in UTMB’s Animal Biosafety Level 4 (ABSL-4) facility at the Galveston National Laboratory. Involved personnel were required to participate in the university’s medical surveillance program. Four- to 6-week-old Hartley guinea pigs (Charles River) were vaccinated intramuscularly on days 0 and 28 with 10 µg of either the WT or prefusion constructs formulated in LNPs. The vaccine was diluted in sterile PBS no more than 24 h prior to administration. The final concentration was 0.05 mg/mL or 10 µg in the 200 µL inoculum. Then, 200 µL of PBS was administered as a mock vaccination. Vaccinations were performed on days 0 and 28. For all blood draws and vaccinations, guinea pigs were anesthetized with 5% isoflurane, and vaccines were administered intramuscularly in two sites in the hind leg with 100 µL per site. Blood was collected in serum separator tubes prior to each vaccination and on day 54 prior to the transfer of the animals to ABSL-4. Blood in 1 mL BD Microtainer serum separation tubes were kept at 4 °C for at least 1 h prior to being spun at 15,000 × *g* for 90 s at room temperature. After being spun down, the serum was collected, aliquoted, and stored at −80 °C. On study day 56, animals were anesthetized with isoflurane and challenged intraperitoneally with 3 × 10^4^ PFU of guinea pig adapted LASV strain Josiah^42^ provided by Dr. Thomas Geisbert and then passaged one additional time in Vero E6 cells. The virus was diluted in sterile PBS to a final volume of 1 mL. Temperature and weight were recorded, and animals were monitored for clinical signs of disease at least daily. Animals that displayed ruffled fur and hunched posture would trigger a score of 2 and two checks per day. Animals that reached a score of 2 and one of the following conditions: lethargy, orbital tightening, or >15% weight loss, triggered a score of 3 and a third check. Finally, animals that reached a score of 3 and one of the following conditions: refusal to stampede, any neurologic signs (seizures, tremors, head tilt, or paralysis), or >20% weight loss were euthanized. Alternatively, for Additional Study 2, animals were euthanized on day 9 to analyze tissues at the expected peak of the viral replication. Sera were titrated on Vero NY cells in 48 well plates to measure viremia. Vero NY cells developed by Dr. Nadya Yun were kindly provided by Dr. Alexander Freiberg. These cells are more permissive to LASV infection, and plaque formation is accelerated.

### Analysis of the binding antibody responses by ELISA

For initial ELISA testing development, the WT post-fusion lysate was used. Due to this antigen being from cell lysate, this assay required subtraction of the background signal, and as such, the output is normalized signal, not specific absorbance (Fig. 2A, B). Our second antigen was a prefusion-stabilized LASV GPC fused to the I53-50A.1NT1 scaffold, generated as described previously^30,31^. GPC-I53-50A.1NT1 proteins of the strains Josiah (clade IV), NIG08-A41(clade II), and Soromba-R (clade V) were diluted to 3 µg/mL in PBS and 50 µL per well was coated on high-binding ELISA plates (Grenier Bio-One). Plates were then covered and incubated at 4 °C overnight. Plates were washed 5 times in PBS with 10% Tween 20 (PBS-T) to remove excess antigen, then blocked for 1 h at 37 °C with PBS-T mixed with 5% skim milk powder (blotto). After blocking, the plates were washed 3 times with PBS-T. Sera were diluted starting at 1:40 in a 4-fold dilution series. Samples were added to the wells, incubated for 30 min, and then plates were washed 6 times as above. The secondary antibody, goat anti-guinea pig heavy and light chain (Kerafast goat anti-guinea pig heavy and light chain; cat#5220-0366) at 1:1000 was applied for 30 min at room temperature, then plates were washed again 6 times. The presence of binding antibodies was visualized with KPL TMB BlueStop Substrate and Stop Solution for 4–6 min. Plates were read at 650 nm on a BioTek plate reader.

### Analysis of LASV-neutralizing antibody responses

Sera were heat-inactivated at 54 °C for 30 min, then diluted in serum-free MEM with 10% guinea pig complement and 0.1% gentamycin sulfate (Corning). A 1:2 dilution series, starting at 1:10, was used. Passage 7, LASV strain Josiah (World Reference Center for Emerging Viruses and Arboviruses, UTMB) was diluted to yield approximately 30 plaques/well and mixed 1:1 with the serum dilutions. This mixture was incubated at 37 °C for 1 h, then transferred to Vero E6 cells (ATCC VERO C1008) in 48 well plates. Virus/serum mixtures were allowed to adsorb onto cells for 1 h at 37 °C, after which they were removed, and an overlay with 0.5% methylcellulose (Spectrum™ methylcellulose, Fisher Scientific) and MEM (Gibco Minimum Essential Media, ThermoFisher Scientific) with 2% FBS (Fetal Bovine Serum, R&D Biosystems) and 0.1% gentamycin (gentamicin sulfate, Corning) was added to each well. Plates were incubated for 3 days and fixed with 10% buffered formalin. Fixed plates were removed from BSL-4, immunostained, and counted. Then, 50% neutralization titers were determined by fitting a regression line.

### Immunostaining

Immunostaining was performed using anti-LASV mouse hyperimmune ascites fluid (HMAF), gifted from Dr. Thomas Ksiazek, diluted to 1:1000 in sterile PBS. After washing the plates with PBS-T, they were blocked in blotto for 1 h at room temperature. After blocking, the plates were incubated with the diluted LASV HMAF for 1 h at room temperature while rocking. Plates were then washed 6 times with PBS-T, and then secondary antibody diluted to 1:1000 was applied for 1 h at room temperature while rocking. The secondary antibody was goat anti-mouse IgG heavy and light chain labeled with horse-radish peroxidase (Seracare, Cat# 5450-0011). The secondary antibodies were dumped off, and the plates were rinsed again 6 times with PBS-T. Plaques were visualized using the AEC Substrate System as per the manufacturer’s recommendations (Abcam, Cat# AB64252). The AEC substrate was left on the plates for 15 min at 37 °C, and then the plates were rinsed in deionized water to stop the reaction. Plates were allowed to dry, and plaques were counted.

### Fc-mediated effector mechanisms

Human neutrophils and NK cells were isolated from fresh peripheral blood. Peripheral blood was collected by the MGH Blood Bank or by the Ragon Institute from healthy volunteers. All volunteers were over 18 years of age and gave signed informed consent. Samples were deidentified before use. The study was approved by the MGH Institutional Review Board.

Antibody-dependent neutrophil phagocytosis (ADNP). White blood cells were isolated from fresh peripheral blood from healthy donors using ammonium-chloride potassium to lyse red blood cells. LASV GP was biotinylated and coupled to yellow-green neutravidin beads, and immune complexes were formed as described for ADCP, using a 1:50 dilution of serum. After incubation, immune complexes were washed, and white blood cells were added to immune complexes at a concentration of 2.5 × 10^5^ cells/mL and incubated for 1 h at 37 °C. Neutrophils were stained with anti-CD66b PacBlue (BioLegend). Fluorescence was measured on an iQue (Intellicyt), and events were gated on singlets, neutrophils (CD66b+) and fluorescent cells. A phagocytic score was calculated as follows: (% bead+ cells)*(MFI of bead+ cells)/10,000.

Antibody-dependent cellular phagocytosis (ADCP). LASV GP (Zalgen Laboratories) was biotinylated using Sulfo-NHS-LC-LC-Biotin. Biotinylated antigen was coupled to yellow-green neutravidin beads (Invitrogen). Immune complexes were formed by adding diluted serum (1:100) to coupled beads and incubating for 2 h at 37 °C. Immune complexes were washed, and THP-1 cells were added to the plates at a concentration of 1.25 × 10^5^ cells/mL. Cells were incubated with immune complexes for 16–18 h at 37 °C. After incubation, fluorescence was measured on an iQue (Intellicyt). Events were gated on singlets and fluorescent cells. A phagocytic score was calculated as described for ADNP.

Antibody-dependent NK cell activation (ADNKA). ELISA plates were coated with antigen at 2 ug/mL and blocked with 5% BSA overnight at 4 °C. NK cells were isolated from fresh peripheral blood from healthy donors using RosetteSep (StemCell Technologies) and separated using a ficoll gradient. NK cells were rested overnight in media supplemented with IL-15. The following day, plates were washed with PBS and serum samples diluted 1:25 were added to the coated plates to form immune complexes. Immune complexes were washed, and NK cells were added at 2.5 × 10^5^ cells/mL in media supplemented with anti-CD107a PE-Cy5 (BD), brefeldin A (Sigma) and GolgiStop (BD). Cells were incubated for 5 h at 37 °C. The cells were stained for surface markers using anti-CD3 PacBlue (BD), anti-CD16 APC-Cy5 (BD) and anti-CD56 PE-Cy7 (BD) and fixed with PermA (Life Tech). NK cells were permeabilized with Perm B (Life Tech) and stained for anti-MIP-1b PE (BD). Fluorescence was acquired using an iQue (Intellicyt) and NK cells were gated as CD3-CD56+CD16+.

Antibody-dependent complement deposition (ADCD). LASV GP was biotinylated and coupled to red neutravidin beads, and immune complexes were formed as described for ADCP, using a 1:10 dilution of serum. Lyophilized guinea pig complement (Cedarlane) was resuspended in cold sterile water and diluted in gelatin veronal buffer with calcium and magnesium (Boston BioProducts). This diluted complement was added to immune complexes, and plates were incubated for 20 min at 37 °C. C3 deposition was stained using anti-C3 FITC (Mpbio). Fluorescence was acquired on an iQue (Intellicyt). Fluorescence is reported as the median fluorescence intensity (MFI) of C3 deposition.

### Mapping of the antibody response by biolayer interferometry

For Biolayer Interferometry antibody competition assays, we used an Octet RED96 (FortéBio) instrument. All samples were diluted to a final volume of 200 µL in 1X kinetics buffer (FortéBio). For the assay, samples were agitated at 1000 RPM at 28 °C in black 96 well plates (Grenier Bio-One, Monroe, NC). We received GPmperP, a biotinylated, non-prefusion stabilized, uncleaved version of GP that contains a linker between GP1 and GP2 from Dr. Hastie. This protein can adopt both the prefusion and postfusion forms. This protein was immobilized onto streptavidin sensors (FortéBio) and then dipped into serum samples for 900 s for saturation. Next, the saturated probes were washed for 60 s two times, and the binding of potentially competing monoclonal antibodies was assessed. The antibodies chosen were 37.7H (Zalgen Labs) specific for the prefusion LASV GPC, 3.3B (Zalgen Labs) specific for LASV GP1, and 22.5D (Zalgen Labs) specific for LASV GP2. The difference in the level of competitor binding to GPmperP was calculated by subtracting the level of competitor mAb binding observed after preincubation with serum from vaccinated animals from the level of mAb binding observed after incubation with serum from non-vaccinated control animals. Data analysis was completed using version 7.0 Octet software.

### Mapping of the antibody response by peptide array

A total of 120 15-mer peptides overlapping by 11 amino acids were designed that covered the length of the LASV strain Josiah GPC. The peptides were produced by JPT Peptide Technologies and immobilized in blocks on glass slides. Then, 1:200 dilutions of day 54 serum samples were incubated for 1 h at 30 °C followed by 4 washes in JPT washing buffer (1× Tris-buffered saline [TBS] buffer [20 mM Tris, 136 mM NaCl, pH 7.4] plus 0.1% Tween 20 [TBS-T]). Naïve guinea pig serum was used as a control. The peptide blocks were then incubated with 0.1 μg/mL anti-guinea pig-Cy5-conjugated antibodies (Jackson ImmunoResearch). The slide was then washed four times with JPT wash buffer and then once with deionized water. The slide was dried via centrifugation and sent to Full Moon Biosystems for recording of fluorescent readings. Fluorescent readings for each spot were then analyzed in-house using GenePix Pro 7 software (Molecular Devices). Heat map from analyzed spots was made in GraphPad.

### Histopathology

Lungs, spleens, and livers were collected from one animal per group for histopathology. After a 24-h incubation at 4 °C, lungs were transferred to fresh 10% formalin for an additional 48-h incubation and removed from BSL-4 containment. Tissues were processed via standard histological procedures by the UTMB Anatomic Pathology Core. Then, 4-μm-thick sections were cut and stained with hematoxylin and eosin. Slides were examined by a trained member of staff.

### Immunohistochemistry

For immunohistochemical analysis, antigen was retrieved from formalin-embedded tissues with EnVision FLEX Target Solution (Agilent Technologies, Cat# GV80411-2) at high PH (Code k8004), at 97 °C for 20 min. Slides were then blocked for 10 min with Dako’s serum-free protein blocking reagent (Agilent Technologies, Cat# X090930-2). Next, the slides were stained with a LASV nucleoprotein rabbit polyclonal antibody (GeneTex, Cat# GTX134883) at 1:4000 for 30 min at room temperature. The slides were then treated with Envision Flex Rabbit Linker at high pH for 15 min (Agilent Technologies, Cat# K800921-2). Next, EnVision HRP was put on the slides for 15 min, followed by EnVision FLEX DAB+ Substrate Chromagen for 5 min (Agilent Technologies, Cat# GV82511-2). Finally, slides were counterstained with Mayer’s Hematoxylin.

### Statistical analysis

Statistical comparisons between groups were made using one-way or two-way ANOVA with post-hoc log-rank, Tukey’s and Mann–Whitney tests (Prism version 9, GraphPad software) as listed in figure legends. *P*-values <0.05 were considered significant.

### Reporting summary

Further information on research design is available in the Nature Portfolio Reporting Summary linked to this article.

### Supplementary information

Supplementary Information

Reporting Summary

### Source data

Source Data

## Data Availability

The data that support this study are available from the corresponding authors upon request. Source data are provided with this paper.

## References

[1] Hallam HJ (2018). Baseline mapping of Lassa fever virology, epidemiology and vaccine research and development. NPJ Vaccines.

[2] Troup JM, White HA, Fom ALMD, Carey DE (1970). An outbreak of Lassa fever on the Jos Plateau, Nigeria, in January–February 1970: a preliminary report. Am. J. Trop. Med. Hyg..

[3] Robinson JE (2016). Most neutralizing human monoclonal antibodies target novel epitopes requiring both Lassa virus glycoprotein subunits. Nat. Commun..

[4] Hastie KM (2017). Structural basis for antibody-mediated neutralization of Lassa virus. Science.

[5] Cross RW (2016). Treatment of Lassa virus infection in outbred guinea pigs with first-in-class human monoclonal antibodies. Antivir. Res..

[6] Sattler RA, Maruyama J, Shehu NY, Makishima T, Paessler S (2019). Current small animal models for LASV hearing loss. Curr. Opin. Virol..

[7] Yun NE, Walker DH (2012). Pathogenesis of Lassa fever. Viruses.

[8] Ter Meulen J (1996). Hunting of peridomestic rodents and consumption of their meat as possible risk factors for rodent-to-human transmission of Lassa virus in the Republic of Guinea. Am. J. Trop. Med. Hyg..

[9] Manning JT, Forrester N, Paessler S (2015). Lassa virus isolates from Mali and the Ivory Coast represent an emerging fifth lineage. Front. Microbiol..

[10] Olayemi, A. & Fichet-Calvet, E. Systematics, ecology, and host switching: attributes affecting emergence of the Lassa virus in rodents across western Africa. *Viruses* **12**, 312 (2020). 10.3390/v12030312 PMC7150792 32183319

[11] Schmitz H (2002). Monitoring of clinical and laboratory data in two cases of imported Lassa fever. Microbes Infect..

[12] Amorosa V (2010). Imported Lassa fever, Pennsylvania, USA, 2010. Emerg. Infect. Dis..

[13] Centers for Disease Control and Prevention (CDC). (2004). Imported Lassa fever—New Jersey, 2004. MMWR Morb. Mortal. Wkly. Rep..

[14] WHO. *Lassa fever—United Kingdom of Great Britain and Northern Ireland*. Disease Outbreak News. https://www.who.int/emergencies/disease-outbreak-news/item/lassa-fever-united-kingdom-of-great-britain-and-northern-ireland (WHO, 2022).

[15] Bell-Kareem, A. R. & Smither, A. R. Epidemiology of Lassa fever. *Curr. Top. Microbiol. Immunol.* **440**, 87–109 (2023). 10.1007/82_2021_234 33861373

[16] Kofman A, Choi MJ, Rollin PE (2019). Lassa fever in travelers from West Africa, 1969–2016. Emerg. Infect. Dis..

[17] Wang M (2021). Construction and immunological evaluation of an adenoviral vector-based vaccine candidate for Lassa fever. Viruses.

[18] Gouglas D, Christodoulou M, Plotkin SA, Hatchett R (2019). CEPI: driving progress toward epidemic preparedness and response. Epidemiol. Rev..

[19] Bernasconi V (2020). Developing vaccines against epidemic-prone emerging infectious diseases. Bundesgesundheitsblatt Gesundheitsforschung Gesundheitsschutz.

[20] CEPI. *Preparing for Lassa Vaccine Trials with Targeted Epidemiology Studies*. Epidemic Preparedness Innovations (CEPI, 2020).

[21] Warner BM, Siragam V, Stein DR (2019). Assessment of antiviral therapeutics in animal models of Lassa fever. Curr. Opin. Virol..

[22] Roberts L (2018). Nigeria hit by unprecedented Lassa fever outbreak. Science.

[23] Siddle KJ (2018). Genomic analysis of Lassa virus during an increase in cases in Nigeria in 2018. N. Engl. J. Med..

[24] Pollard C, De Koker S, Saelens X, Vanham G, Grooten J (2013). Challenges and advances towards the rational design of mRNA vaccines. Trends Mol. Med..

[25] Kariko K, Buckstein M, Ni H, Weissman D (2005). Suppression of RNA recognition by Toll-like receptors: the impact of nucleoside modification and the evolutionary origin of RNA. Immunity.

[26] Anderson BR (2010). Incorporation of pseudouridine into mRNA enhances translation by diminishing PKR activation. Nucleic Acids Res..

[27] Andries O (2015). N^1^-methylpseudouridine-incorporated mRNA outperforms pseudouridine-incorporated mRNA by providing enhanced protein expression and reduced immunogenicity in mammalian cell lines and mice. J. Control Release.

[28] Kariko K (2008). Incorporation of pseudouridine into mRNA yields superior nonimmunogenic vector with increased translational capacity and biological stability. Mol. Ther..

[29] Haynes BF (2021). A new vaccine to battle Covid-19. N. Engl. J. Med..

[30] Brouwer PJM (2022). Lassa virus glycoprotein nanoparticles elicit neutralizing antibody responses and protection. Cell Host Microbe.

[31] Perrett, H. R. et al. Structural conservation of Lassa virus glycoproteins and recognition by neutralizing antibodies. *Cell Rep.* **42**, 112524 (2023). 10.1016/j.celrep.2023.112524 PMC10242449 37209096

[32] Mire CE (2017). Human-monoclonal-antibody therapy protects nonhuman primates against advanced Lassa fever. Nat. Med..

[33] Safronetz D (2015). The broad-spectrum antiviral favipiravir protects guinea pigs from lethal Lassa virus infection post-disease onset. Sci. Rep..

[34] Jahrling PB (1980). Lassa virus infection of rhesus monkeys: pathogenesis and treatment with ribavirin. J. Infect. Dis..

[35] Bell TM (2017). Temporal progression of lesions in guinea pigs infected with Lassa virus. Vet. Pathol..

[36] Painter MM (2021). Rapid induction of antigen-specific CD4^+^ T cells is associated with coordinated humoral and cellular immunity to SARS-CoV-2 mRNA vaccination. Immunity.

[37] Carrion R (2007). A ML29 reassortant virus protects guinea pigs against a distantly related Nigerian strain of Lassa virus and can provide sterilizing immunity. Vaccine.

[38] Carnec, X. et al. A vaccine platform against arenaviruses based on a recombinant hyperattenuated Mopeia virus expressing heterologous glycoproteins. *J. Virol.* **92**, e02230-17 (2018). 10.1128/JVI.02230-17 PMC5974477 29593043

[39] Cashman KA (2017). A DNA vaccine delivered by dermal electroporation fully protects cynomolgus macaques against Lassa fever. Hum. Vaccin. Immunother..

[40] Jiang J (2019). Immunogenicity of a protective intradermal DNA vaccine against Lassa virus in cynomolgus macaques. Hum. Vaccin. Immunother..

[41] Cross RW (2020). Quadrivalent VesiculoVax vaccine protects nonhuman primates from viral-induced hemorrhagic fever and death. J. Clin. Invest..

[42] Geisbert TW (2005). Development of a new vaccine for the prevention of Lassa fever. PLoS Med..

[43] Mateo M (2019). Vaccines inducing immunity to Lassa virus glycoprotein and nucleoprotein protect macaques after a single shot. Sci. Transl. Med..

[44] Abreu-Mota T (2018). Non-neutralizing antibodies elicited by recombinant Lassa-Rabies vaccine are critical for protection against Lassa fever. Nat. Commun..

[45] Watts G (2012). Lily Lyman Pinneo. Lancet.

[46] Cross RW (2019). Antibody therapy for Lassa fever. Curr. Opin. Virol..

[47] Hastie KM (2019). Convergent structures illuminate features for germline antibody binding and pan-Lassa virus neutralization. Cell.

[48] Heinrich ML (2020). Antibodies from Sierra Leonean and Nigerian Lassa fever survivors cross-react with recombinant proteins representing Lassa viruses of divergent lineages. Sci. Rep..

[49] McLellan JS (2013). Structure of RSV fusion glycoprotein trimer bound to a prefusion-specific neutralizing antibody. Science.

[50] Cashman KA (2013). Enhanced efficacy of a codon-optimized DNA vaccine encoding the glycoprotein precursor gene of Lassa virus in a guinea pig disease model when delivered by dermal electroporation. Vaccines.

[51] Kainulainen MH (2018). Use of a scalable replicon-particle vaccine to protect against lethal Lassa virus infection in the guinea pig model. J. Infect. Dis..

[52] Kennedy EM (2019). A vaccine based on recombinant modified Vaccinia Ankara containing the nucleoprotein from Lassa virus protects against disease progression in a guinea pig model. Vaccine.

[53] Banasik BN (2019). Development of an anti-guinea pig CD4 monoclonal antibody for depletion of CD4+ T cells in vivo. J. Immunol. Methods.

[54] Thomas, S. J. et al. Safety and efficacy of the BNT162b2 mRNA Covid-19 vaccine through 6 months. *N. Engl. J. Med.* **385**, 1761–1773 (2021). 10.1056/NEJMoa2110345 PMC8461570 34525277

[55] Baden LR (2020). Efficacy and safety of the mRNA-1273 SARS-CoV-2 vaccine. N. Engl. J. Med..

[56] Meyer M (2018). Modified mRNA-based vaccines elicit robust immune responses and protect guinea pigs from Ebola virus disease. J. Infect. Dis..

[57] Nelson J (2020). Impact of mRNA chemistry and manufacturing process on innate immune activation. Sci. Adv..

[58] Hassett KJ (2019). Optimization of lipid nanoparticles for intramuscular administration of mRNA vaccines. Mol. Ther. Nucleic Acids.

