# Supplementary material for: A Lassa virus mRNA vaccine confers protection but does not require neutralizing antibody in a guinea pig model of infection
Source: Nat Commun. 2023 Sep 12;14:5603. doi: 10.1038/s41467-023-41376-6 (PMC10497546; doi:10.1038/s41467-023-41376-6)
Supplement: Supplementary file 1 — Supplementary Information [file 41467_2023_41376_MOESM1_ESM.pdf]

A

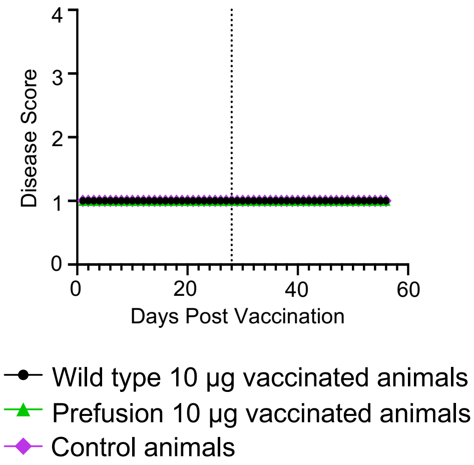

B

Weight (g)			
Wild type vaccinated animals			
Animal	Day -1	Day 28	Day 54
M1-W-10-1	335	485	570
M1-W-10-2	340	535	645
M1-W-10-3	355	490	555
M1-W-10-4	365	500	585
M1-W-10-5	355	490	555
Prefusion vaccinated animals			
Animal	Day -1	Day 28	Day 54
M1-P-10-1	365	490	545
M1-P-10-2	360	530	630
M1-P-10-3	360	475	550
M1-P-10-4	370	540	655
M1-P-10-5	350	470	555

Supplementary Figure 1. Clinical data for vaccinated animals before the LASV challenge. **A.** Disease scores. The dashed line indicates day 28, where vaccinated animals received a second dose of vaccine or PBS. **B.** Animal weights on one day prior to first vaccination, day 28, and day 54 post vaccination. For all figures, WT GPC vaccinated animals represented by a filled-in black circle. Prefusion GPC vaccinated animals represented by a filled-in green triangle. All non-vaccinated control animals represented but a filled-in purple diamond.

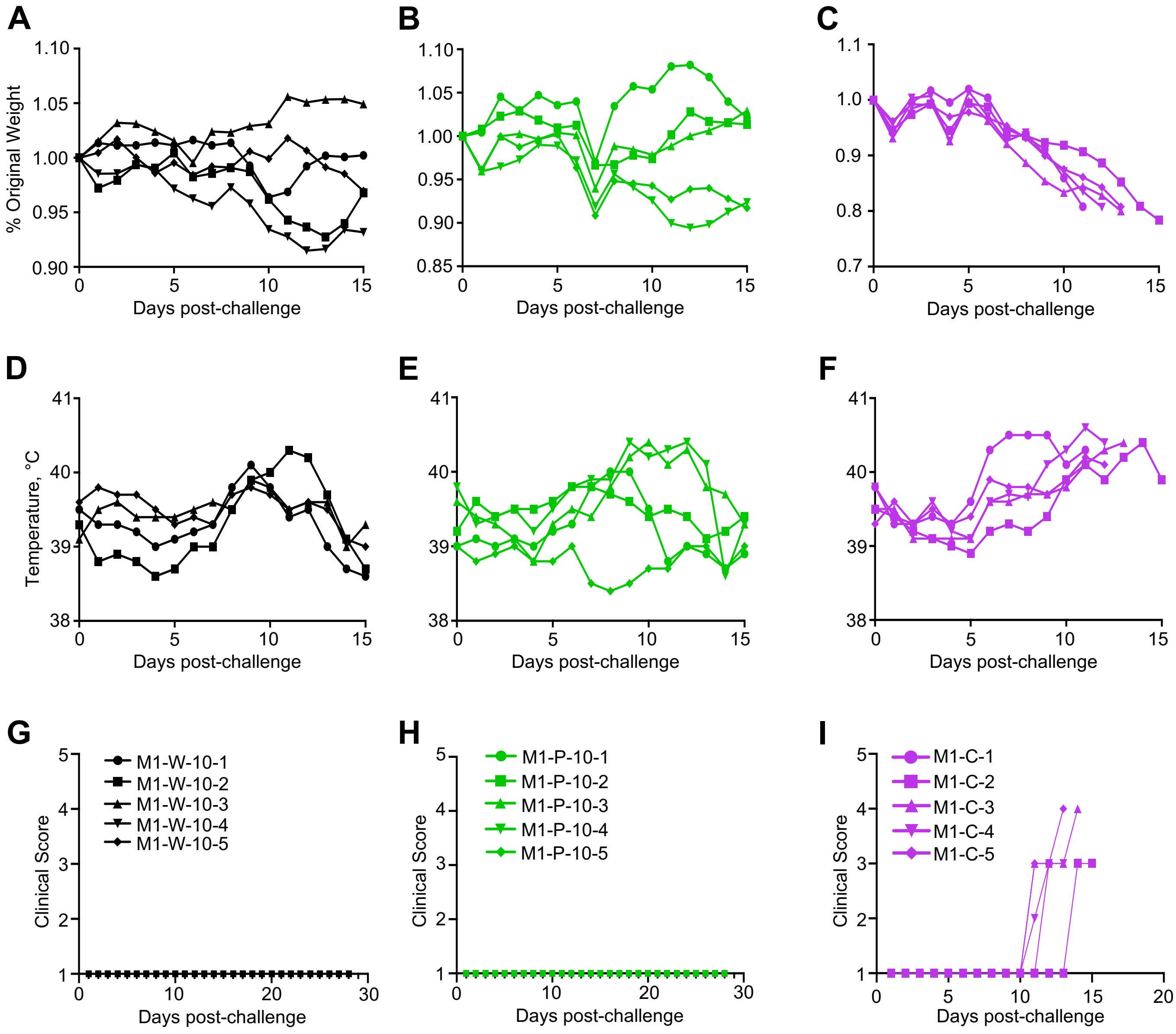

Supplementary Figure 2. Disease parameters for individual study animals. A. Percent of original weight in animals vaccinated with 10 µg of the wild type vaccine construct. **B.** Percent of original weight in animals vaccinated with 10 µg of the prefusion vaccine construct. **C.** Percent of original weight in LASV challenged control animals. **D.** Temperature change from days 0-15 post-challenge for animals vaccinated with 10 µg of the wild type vaccine construct. This chart is missing values from animal M1-W-10-4, as the temperature transponder chip was broken. **E.** Temperature change from days 0-15 post-challenge for animals vaccinated with 10 µg of the prefusion vaccine construct. **F.** Temperature change from days 0-15 post-challenge for LASV challenged control animals. **G**. Clinical scores for animals vaccinated with the wildtype vaccine construct. **H**. Clinical scores for animals vaccinated with the prefusion vaccine construct. **I.** Clinical scores for the challenged control animals. For all figures, WT GPC vaccinated animals are represented by the shapes in black – A, D, and G. Prefusion GPC vaccinated animals are represented by shapes in green – B, E, and H. Control, non-vaccinated animals are represented by shapes in purple – C, F, and I.

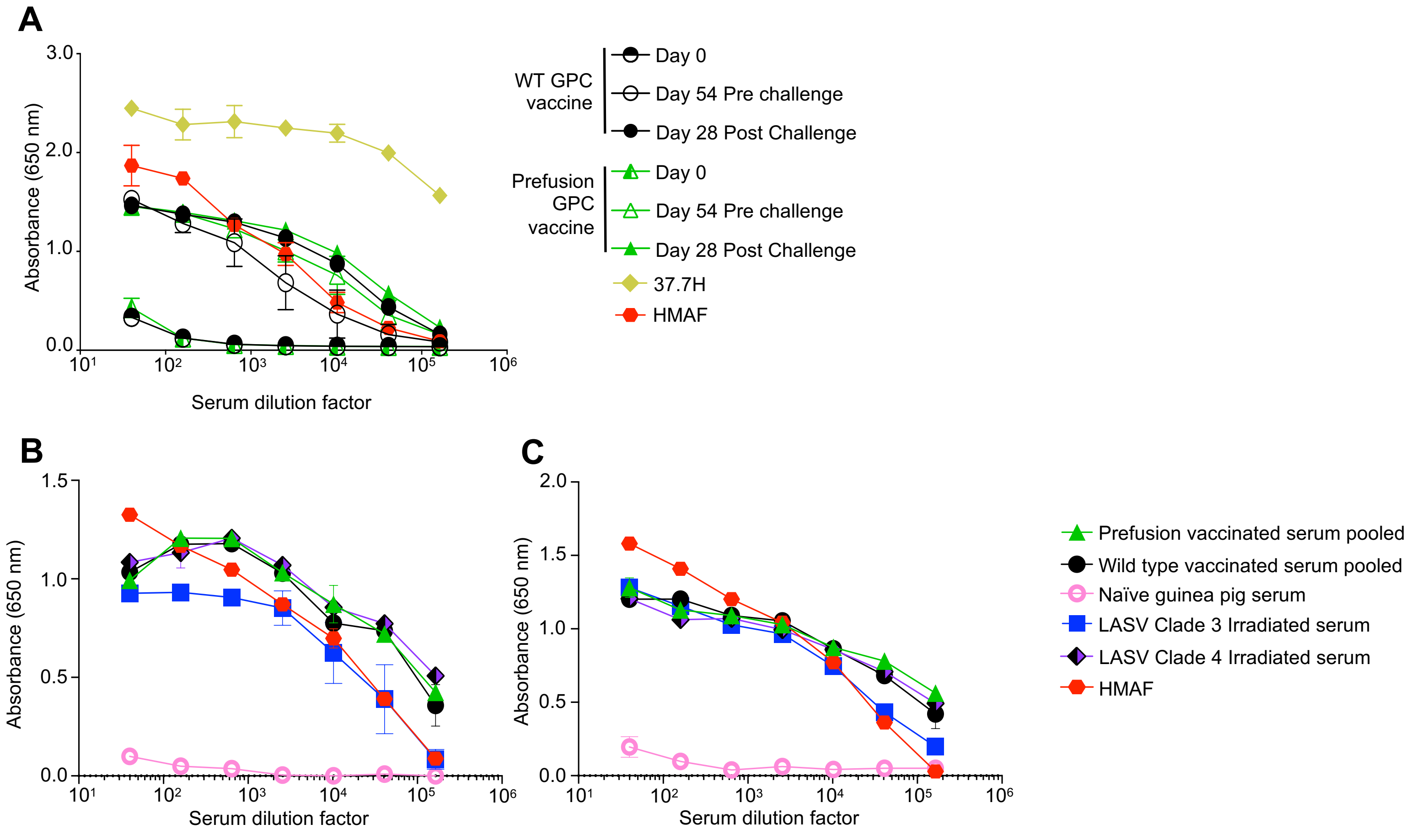

Supplementary Figure 3. Quantification of IgG binding antibodies post-challenge to Lassa strain Josiah GPC and two forms of LASV strain Josiah NP protein. A. Binding of IgG binding from day 0, day 54, and pooled post challenge serum to prefusion stabilized LASV GPC determined by ELISA. N=5. Each timepoint is an average of the 5 animals per vaccine group. For figure A, WT GPC vaccinated animals are represented by a filled-in black circle. Prefusion GPC vaccinated animals are represented by a filled-in green triangle. Antibody control, 37.7H is represented by a mustard-colored diamond. Serum control, HMAF, is represented by a red hexagon. **B**. Full length NP protein (Zalgen, LASV-R-0041). **C.** NP N-terminus amino acids 1-340, (Zalgen, LASV-R-0042). For each ELISA, samples were analyzed in duplicates, and the assays were performed twice. Duplicate values were averaged, and the error bars represent the standard deviation between the duplicate values. Prefusion and wild type vaccinated sera were pooled from the 10 wild type and 10 prefusion vaccinated animals, respectively. For figures B and C, prefusion vaccinated serum is represented by a filled, green triangle. Wild type vaccinated serum is represented but a filled, black circle. Naïve, uninfected guinea pig serum is represented by an empty, pink circle. LASV clade 3 serum is represented by a blue square. LASV clade 4 serum is represented by a half black and half purple diamond. HMAF, a serum control, is represented by a red hexagon.

Additional Study 1: Liver, collected on days 15-28 post challenge

A

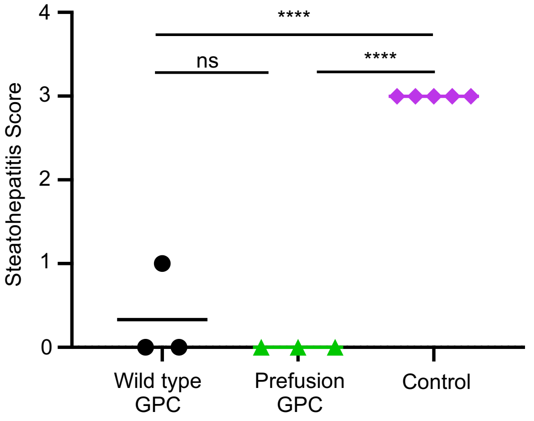

B

Grading and staging for NAFLD/NASH		
**Component Scores**		
**Steatosis grade**	**Lobular inflammation**	**Hepatocellular ballooning**
0: <5%	0: None	0: None
1: 5-33%	1:<2 foci/x20 field	1: Mild, few
2: 34-66%	2: 2-4 foci/x20 field	2: moderate-marked, many
3: >66%	3: >4 foci/x20 field	
**NAFLD activity score (NAS): 0-8**		
Steatosis (0-3) + Lobular inflammation (0-3) + Ballooning (0-2)		

C

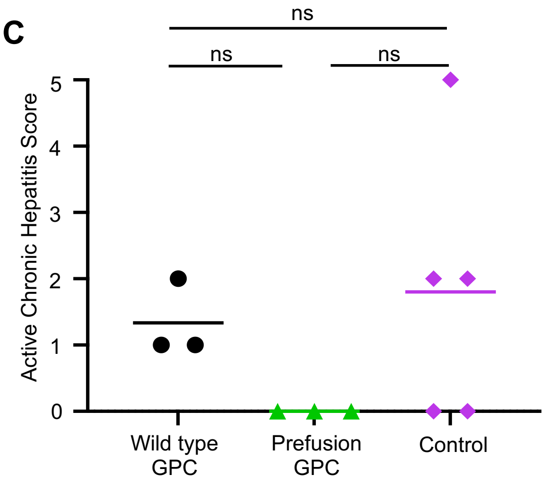

D

ACTIVE CHRONIC HEPATITIS SCORE	
**A. Periportal or periseptal interface hepatitis (piecemeal necrosis)**	
Absent	0
Mild (focal, few portal areas)	1
Mild/moderate (focal, most portal areas)	2
Moderate (continuous around ~50% of tracts or septa)	3
Severe (continuous around ~50% of tracts or septa)	4
**B. Confluent necrosis**	
Absent	0
Focal confluent necrosis	1
Zone 3 necrosis in some areas	2
Zone 3 necrosis in most areas	3
Zone 3 necrosis + occasional portal-central (P-C) bridging	4
Zone 3 necrosis + multiple P-C bridging	5
Panacinar or multiacinar necrosis	6
**C. Focal (spotty) lytic necrosis, apoptosis, and focal inflammation**	
Absent	0
1 focus or less per 10x objective	1
2 to 4 foci per 10x objective	2
5 to 10 foci per 10x objective	3
More than 10 foci per 10x objective	4
**D. Portal inflammation**	
None	0
Mild, some or all portal areas	1
Moderate, some or all portal areas	2
Moderate/marked, all portal areas	3
Marked, all portal areas	4

Supplementary Figure 4. Grading of liver pathology of vaccinated and non-vaccinated animals. A. Steatohepatitis scores for individual animals per vaccine group at day 28 post challenge. Three animals in wild type GPC group and prefusion stabilized GPC group each. Five animals in the control group. One-way ANOVA analysis with Brown-Forsyth test in Prism 9. **** p<0.0001. ns, p=0.3789 **B**. Grading and staging for non-alcoholic fatty liver disease/non-alcoholic steatohepatitis (NAFLD/NASH). Descriptions of the component scores for steatohepatitis scores. Adapted from Knodell RG., et al. Formulation and scoring system for assessing histological activity in asymptomatic chronic active hepatitis. 1(5):431-435. 1981. PMID: 7308988, DOI: 10.1002/hep.1840010511. **C.** Active chronic hepatitis scores for individual animals by vaccine group at day 28 post challenge. Three animals in wild type GPC group and prefusion stabilized GPC group each. Five animals in the control group. One-way ANOVA analysis with Brown-Forsyth test in Prism version 9. Between WT and PreF ns, p= 0.5375. Between WT and control ns, p=0.9032. Between PreF and control ns, p=0.2745. **D**. Description of the component scores for the Active Chronic Hepatitis Score. Adapted from Ishak K. et al. Histological grading and staging of chronic hepatitis. J hepatol. 22:696-699. 1995. PMID: 756085, DOI: 10.1016/0168-8278(95)80226-6. For all figures, WT GPC vaccinated animals represented by a filled-in black circle. Prefusion GPC vaccinated animals represented by a filled-in green triangle. All non-vaccinated control animals represented but a filled-in purple diamond.

Additional Study 2: Lung, collected on day 9 post challenge

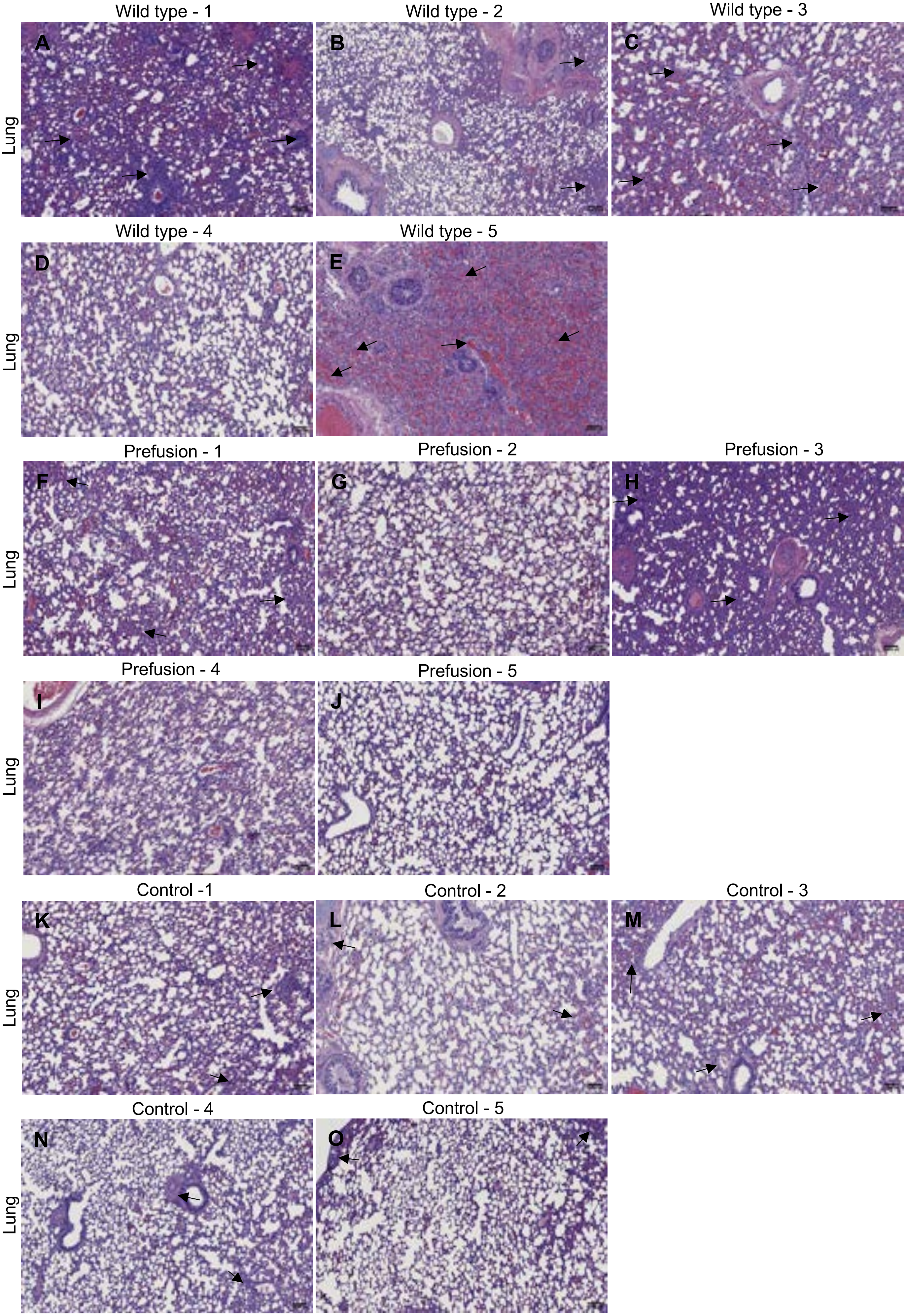

Supplementary Figure 5. Histopathology of vaccinated and non-vaccinated LASV challenged guinea pig lungs collected at day 9 post challenge. All tissues were stained with H&E. **A-E:** Animals were vaccinated with two 10 µg doses of the wild type GPC construct. **F-J:** Animals were vaccinated with two 10 µg doses of the the prefusion stabilized GPC construct. **K-O:** Unvaccinated control animals. Arrows indicate areas of interstitial pneumonia and septal thickening, and in one wild type animal intra-alveolar hemorrhage.

Additional Study 1: Spleen, collected on days 15-28 post challenge

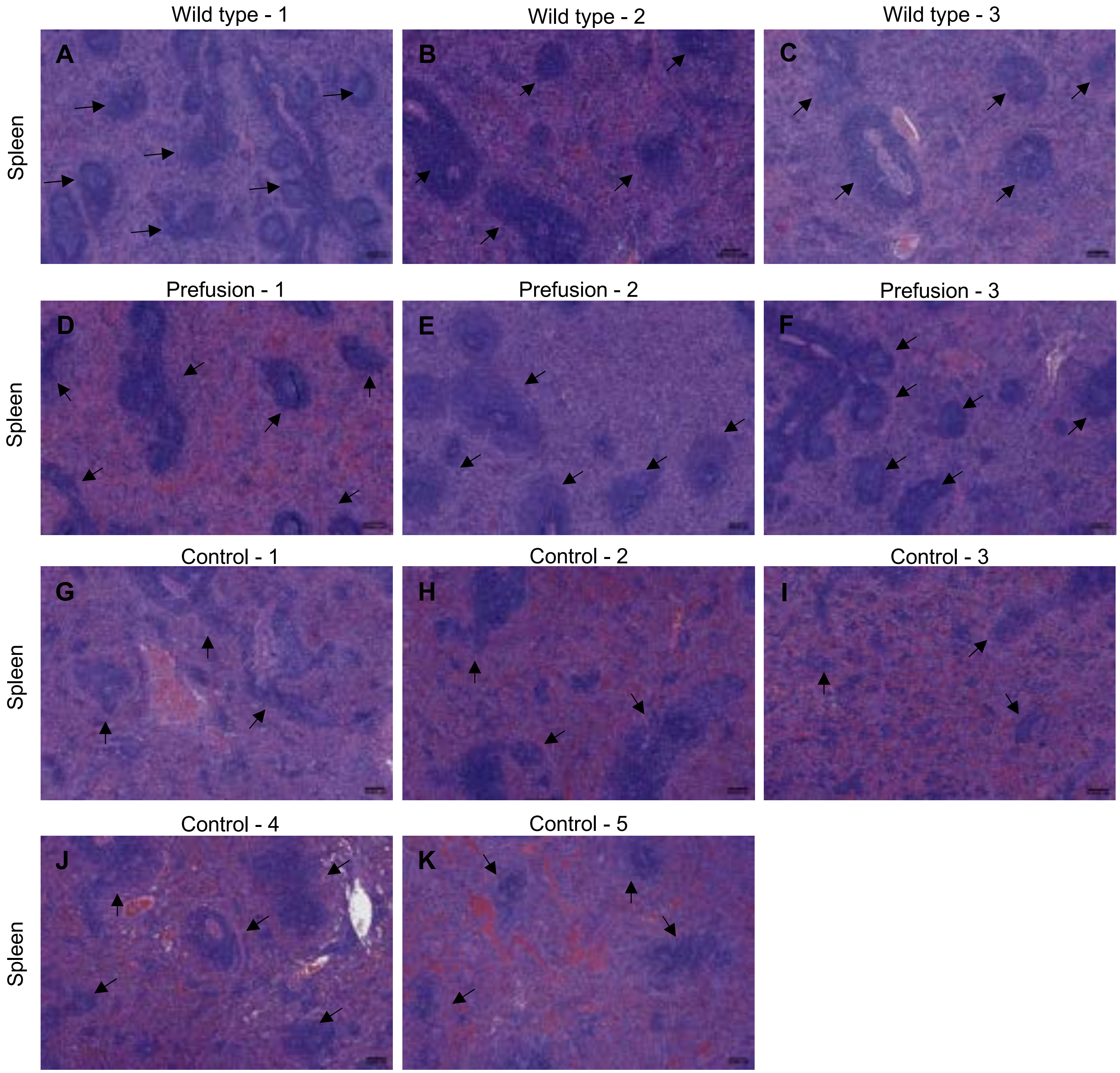

Supplementary Figure 6. Histopathology of vaccinated and non-vaccinated LASV challenged guinea pig spleens collected at day 28 post challenge. All tissues stained with H&E. **A-C:** Animals were vaccinated with two 10 µg doses of the of wild type GPC construct. **D-F:** Animals were vaccinated with two 10 µg doses of the prefusion stabilized GPC construct. **G-K:** Unvaccinated control animals. Arrows indicate areas of germinal center activation.

Additional Study 2: Spleen, collected on day 9 post challenge

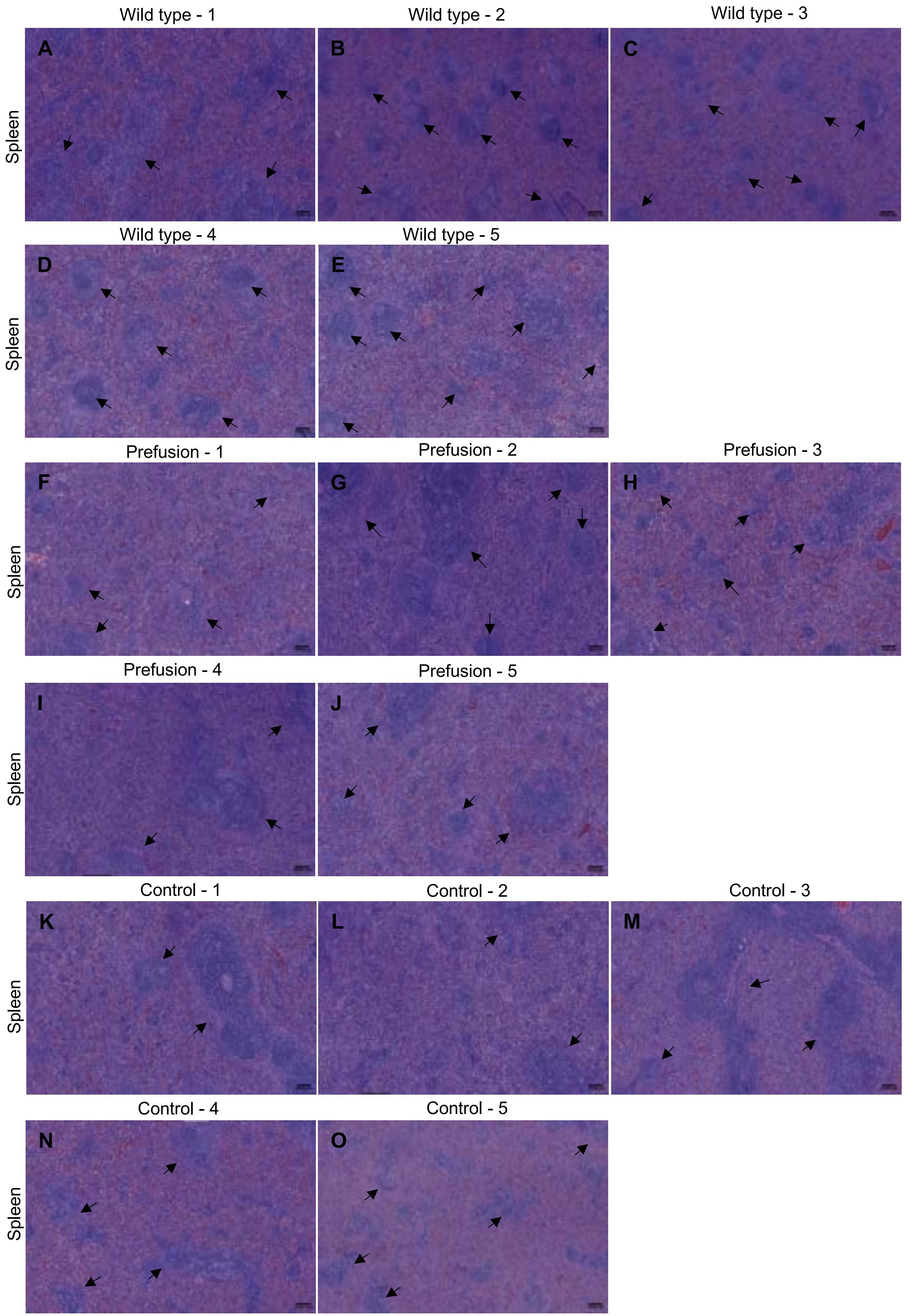

Supplementary Figure 7. Histopathology of vaccinated and non-vaccinated LASV challenged guinea pig spleens collected at day 9 post challenge. A-E: Animals were vaccinated with two 10 μg doses of the wild type GPC construct. **F-J** were vaccinated with two 10 μg doses of the prefusion stabilized GPC construct. **K-O:** Unvaccinated control animals. Arrows indicate areas of germinal center activation. All spleen pathology was observed to be normal.

Additional Study 1: Liver, collected on days 15-28 post challenge

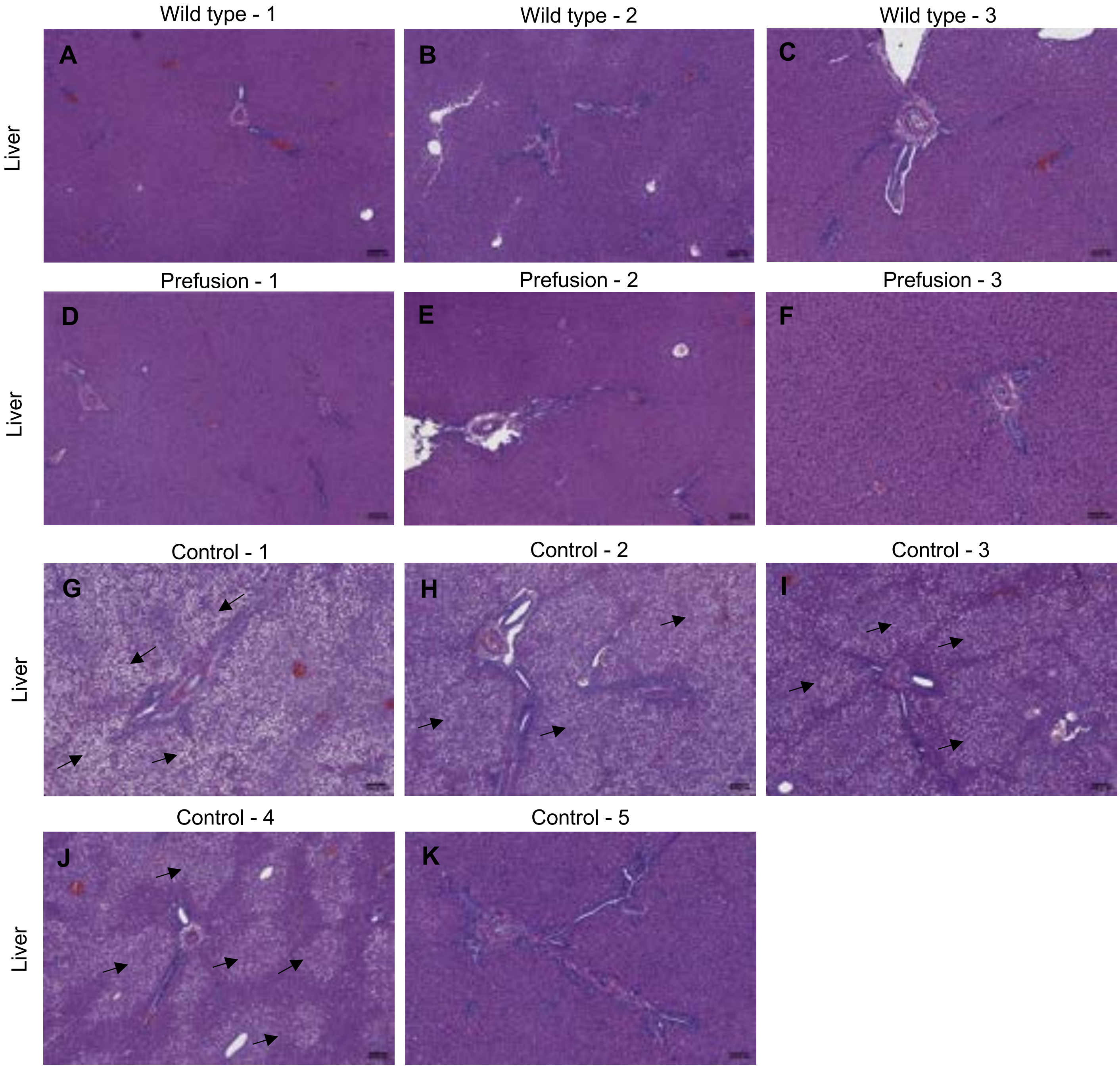

Supplementary Figure 8. Histopathology of vaccinated and non-vaccinated LASV challenged guinea pig livers collected at day 28 post challenge. All tissues stained with H&E. **A-C:** Animals were vaccinated with two 10 μg doses of the of wild type GPC construct. **D-F:** Animals were vaccinated with two 10 μg doses of the prefusion stabilized GPC construct. **G-K:** Unvaccinated control animals. Black arrows indicate areas with notable steatosis. As expected, there was no significant change in pathology in vaccinated animals.

Additional Study 2: Liver, collected at day 9 post challenge

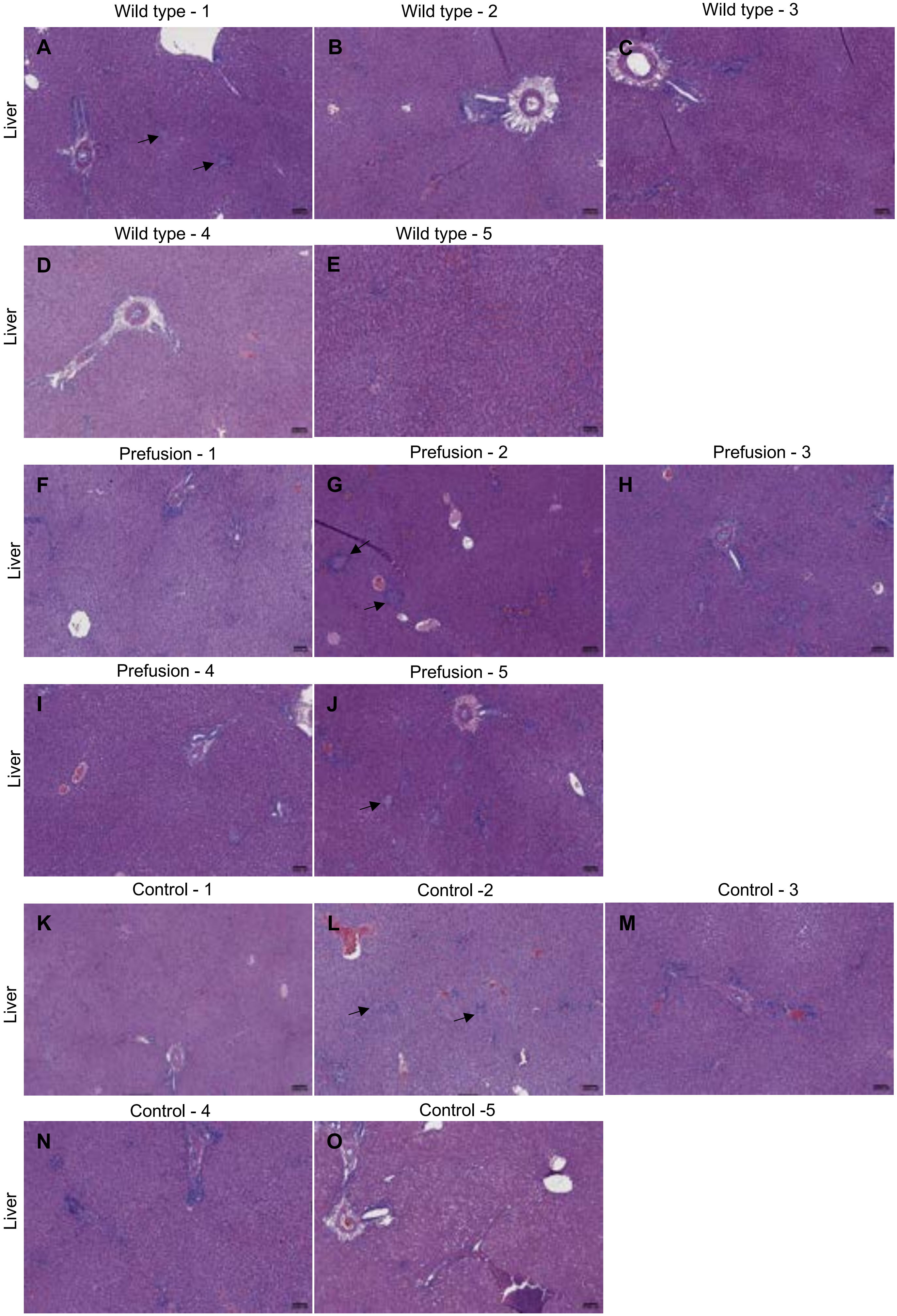

Supplementary Figure 9. Histopathology of vaccinated and non-vaccinated LASV challenged guinea pig livers collected at day 9 post challenge. All tissues were stained with H&E. **A-E:** Animals were vaccinated with two 10 µg doses of the wild type GPC construct. **F-J** were vaccinated with two 10 µg doses of the prefusion stabilized GPC construct. **K-O:** Unvaccinated control animals. Due to the early time post challenge, liver tissue was deemed mostly normal. Some spotty lytic necrosis was noted at 1 focus or less per 10x objective, but otherwise the tissue was unremarkable.

Additional Study 1: Spleen, immunohistochemistry, collected on days 15-28 post challenge

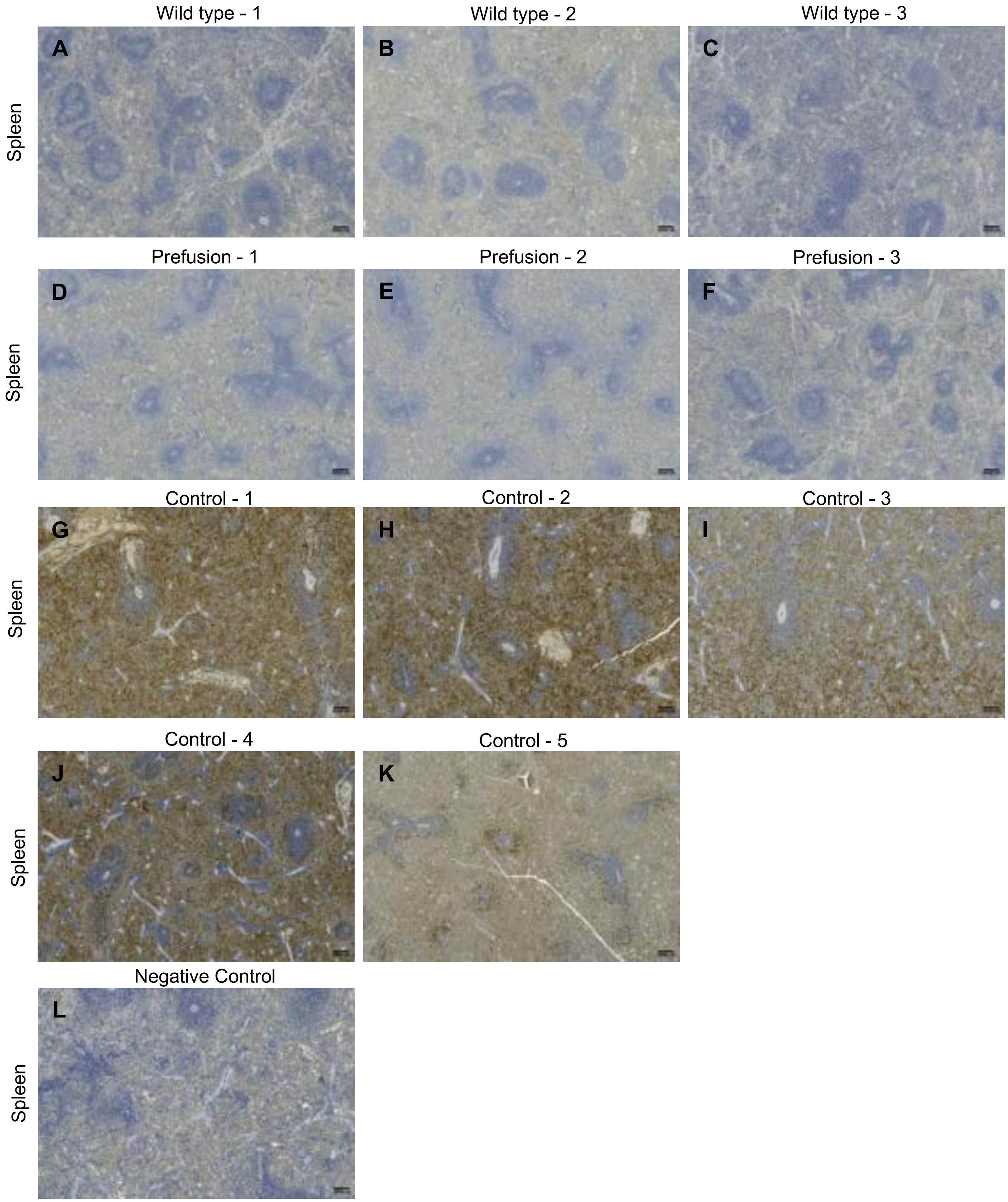

Supplementary Figure 10. Histopathology of vaccinated and non-vaccinated LASV challenged guinea pig spleens collected at day 28 post challenge. All tissues were immunostained with a LASV nucleoprotein antibody. **A-C:** Animals were vaccinated with two 10 µg doses of wild type GPC construct. **D-F:** Animals were vaccinated with two 10 µg doses of the prefusion stabilized GPC construct. **G-K:** Unvaccinated control animals. **L:** Naïve, negative control tissue. As expected, there was no significant staining in vaccinated animals.

Additional Study 1: Liver, immunohistochemistry, collected on days 15-28 post challenge

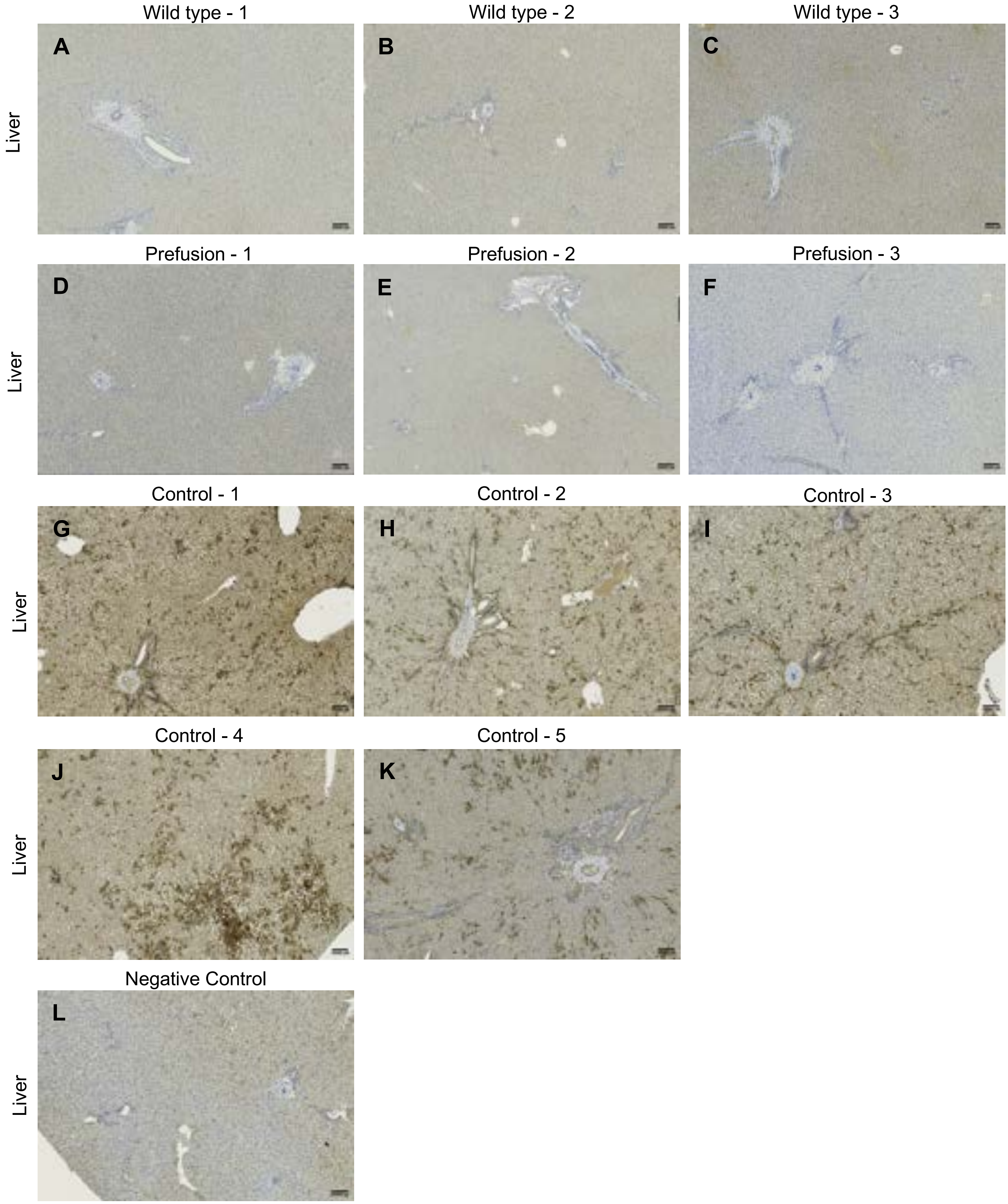

Supplementary Figure 11. Histopathology of vaccinated and non-vaccinated LASV challenged guinea pig livers collected at day 28 post challenge. All tissues were immunostained with a LASV nucleoprotein antibody. **A-C:** Animals were vaccinated with two 10 µg doses of the of wild type GPC construct. **D-F:** Animals were vaccinated with two 10 µg doses of the prefusion stabilized GPC construct. **G-K:** Unvaccinated control animals. **L:** Naïve, negative control tissue. As expected, there was no significant staining in vaccinated animals.

Additional Study 2: Lung, collected on day 9 post challenge

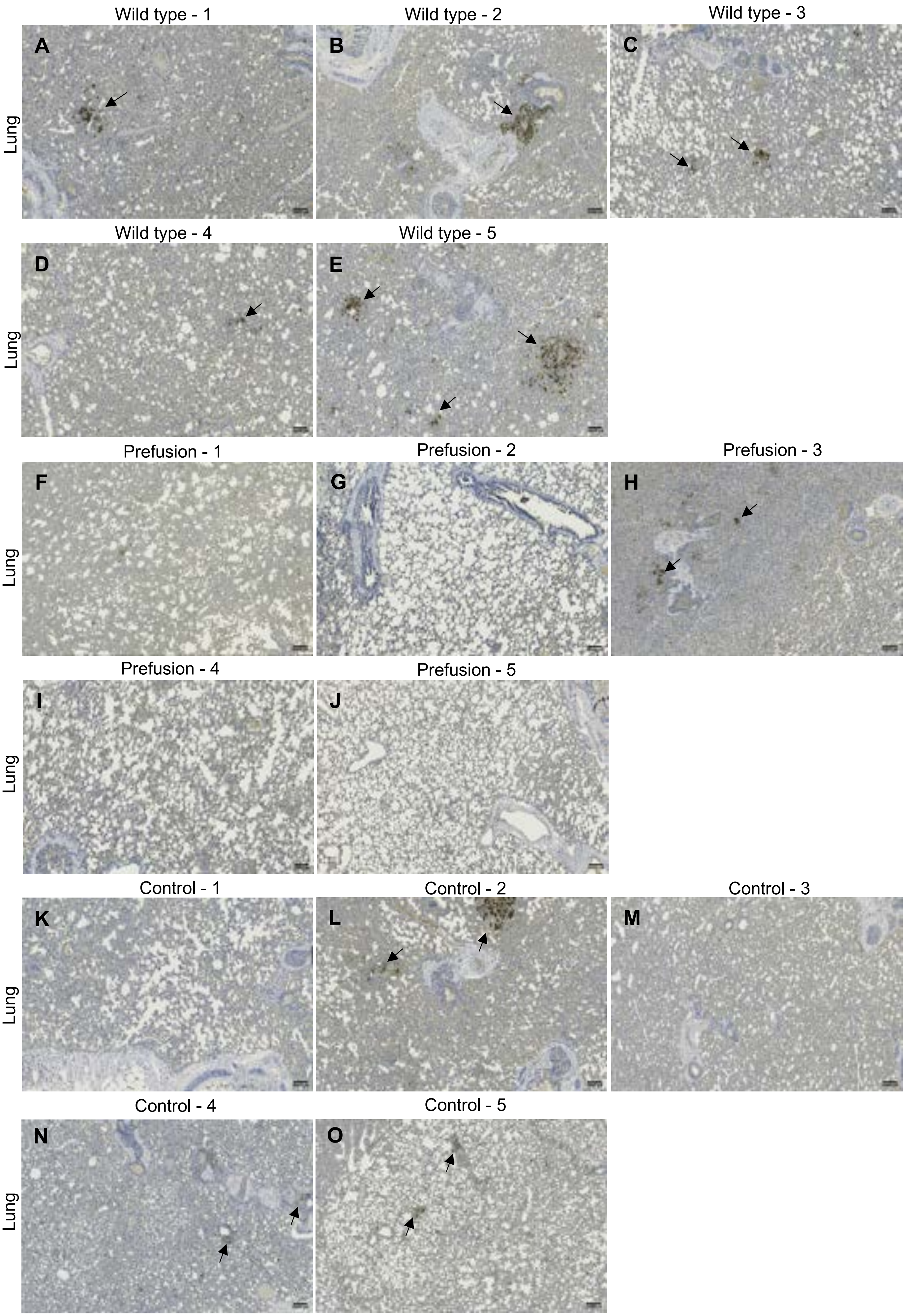

Supplementary Figure 12. Immunohistochemistry of vaccinated and non-vaccinated LASV challenged guinea pig lungs collected at day 9 post challenge. All tissues were immunostained with a LASV nucleoprotein antibody. **A-E:** Animals were vaccinated with two 10 µg doses of the wild type GPC construct. **F-J:** Animals were vaccinated with two 10 µg doses of the prefusion stabilized GPC construct. **K-O:** Unvaccinated control animals. Arrows indicate areas of notable NP staining.

Additional Study 2: Spleen, collected on day 9 post challenge

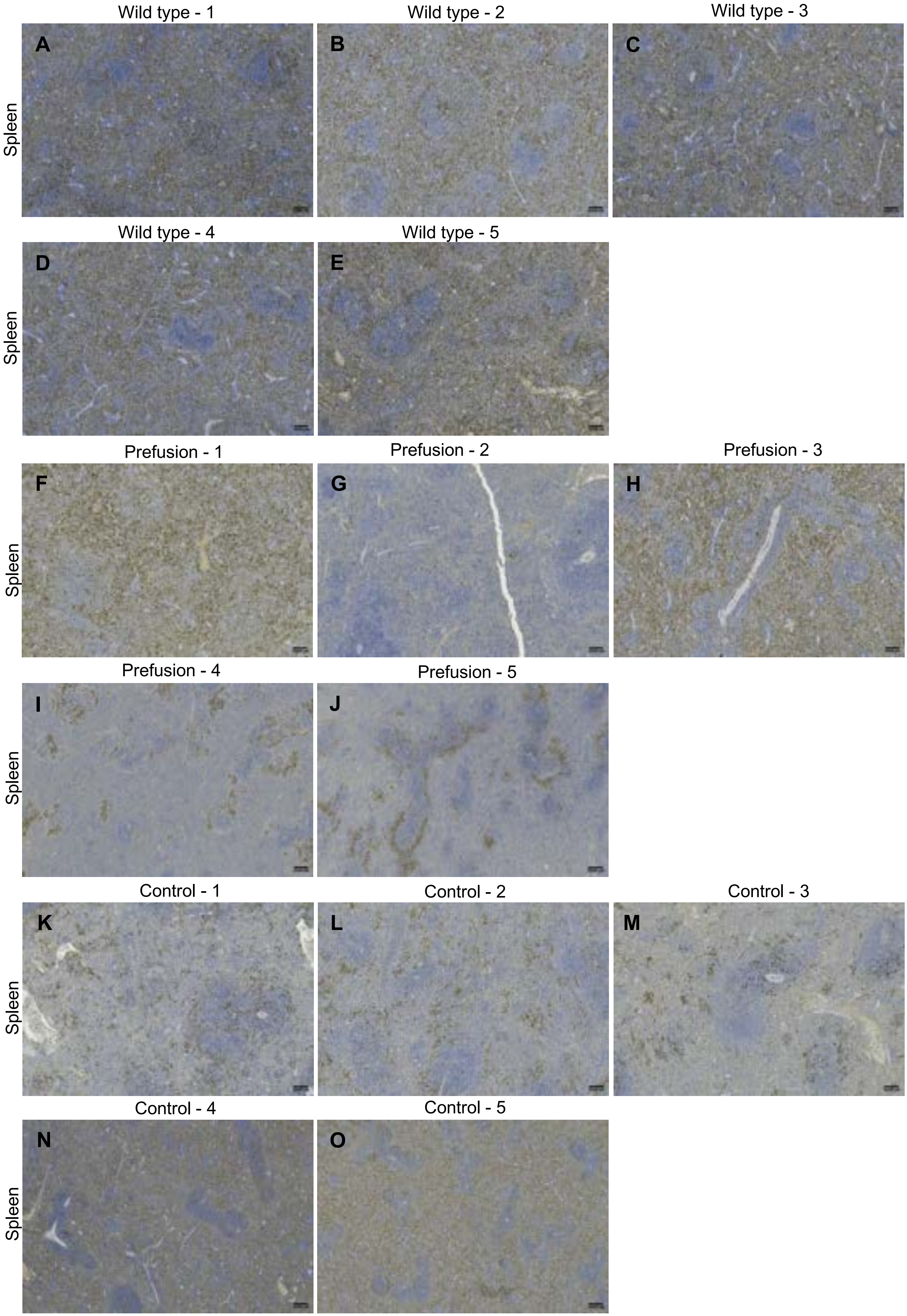

Supplementary Figure 13. Immunohistochemistry of vaccinated and non-vaccinated LASV challenged guinea pig spleens collected at day 9 post challenge. All tissues were immunostained with a LASV nucleoprotein antibody. **A-E:** Animals were vaccinated with two 10 µg doses of the wild type GPC construct. **F-J:** Animals were vaccinated with two 10 µg doses of the prefusion stabilized GPC construct. **K-O:** Unvaccinated control animals. Staining was positive in all tissues.

Additional Study 2: Liver, collected at day 9 post challenge

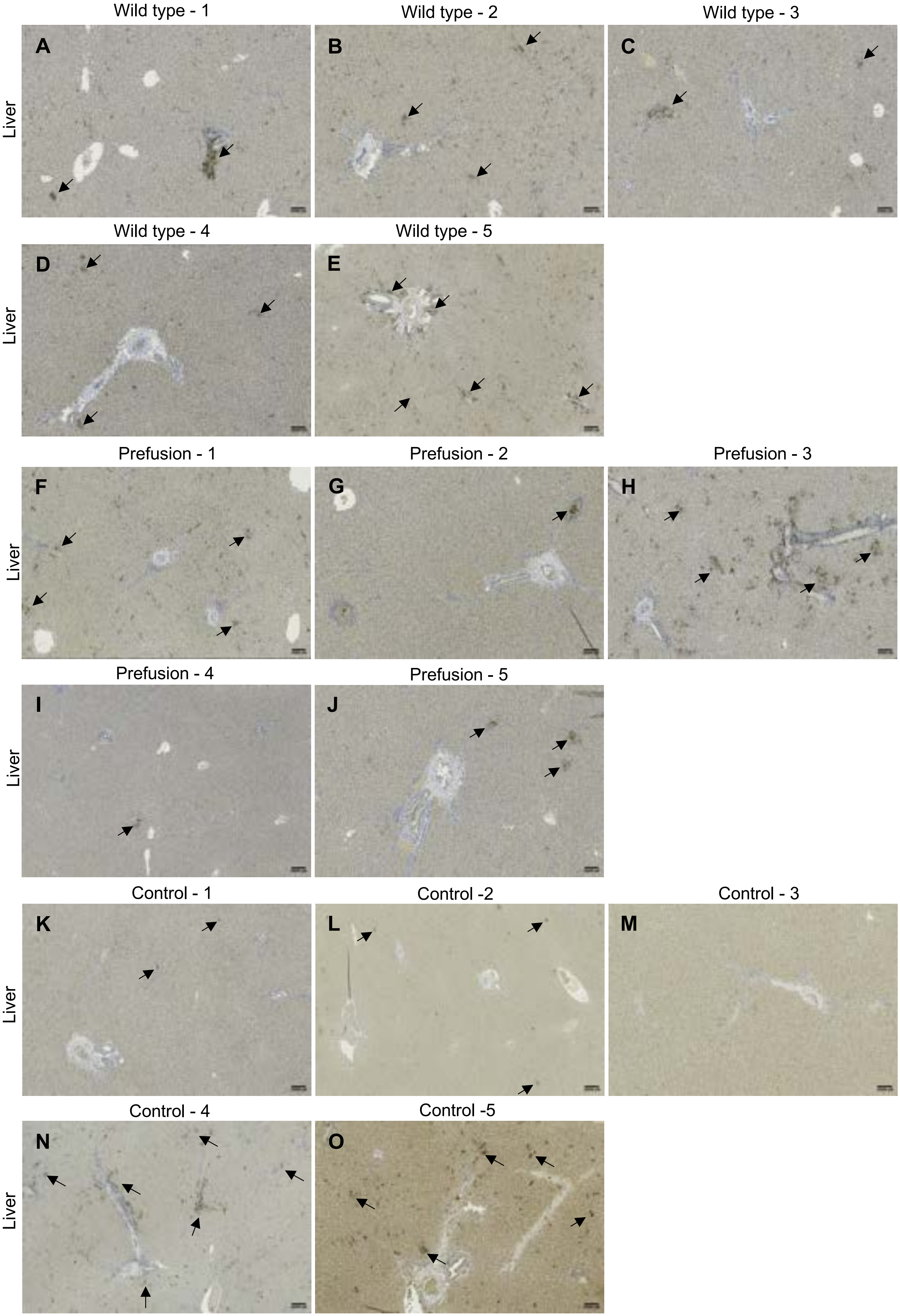

Supplementary Figure 14. Immunohistochemistry of vaccinated and non-vaccinated LASV challenged guinea pig livers collected at day 9 post challenge. All tissues were immunostained with a LASV nucleoprotein antibody. **A-E:** Animals were vaccinated with two 10 µg doses of the wild type GPC construct. **F-J:** Animals were vaccinated with two 10 µg doses of the prefusion stabilized GPC construct. **K-O:** Unvaccinated control animals. Arrows indicate areas of notable NP staining. All tissues but Control-3 were positive for NP staining.
