# Supplementary material for: A Lassa virus mRNA vaccine confers protection but does not require neutralizing antibody in a guinea pig model of infection
Source: Nat Commun. 2023 Sep 12;14:5603. doi: 10.1038/s41467-023-41376-6 (PMC10497546; doi:10.1038/s41467-023-41376-6)
Supplement: Supplementary file 2 — Reporting Summary [file 41467_2023_41376_MOESM2_ESM.pdf]

nature portfolio

Corresponding author(s): Alexander Bukreyev; Andrea Carfi

Last updated by author(s): Aug 28, 2023

Reporting Summary

Nature Portfolio wishes to improve the reproducibility of the work that we publish. This form provides structure for consistency and transparency in reporting. For further information on Nature Portfolio policies, see our Editorial Policies and the Editorial Policy Checklist.

Statistics

For all statistical analyses, confirm that the following items are present in the figure legend, table legend, main text, or Methods section.

n/a	Confirmed	
☐	☒	The exact sample size (*n*) for each experimental group/condition, given as a discrete number and unit of measurement
☐	☒	A statement on whether measurements were taken from distinct samples or whether the same sample was measured repeatedly
☐	☒	The statistical test(s) used AND whether they are one- or two-sided<br>*Only common tests should be described solely by name; describe more complex techniques in the Methods section.*
☒	☐	A description of all covariates tested
☒	☐	A description of any assumptions or corrections, such as tests of normality and adjustment for multiple comparisons
☐	☒	A full description of the statistical parameters including central tendency (e.g. means) or other basic estimates (e.g. regression coefficient) AND variation (e.g. standard deviation) or associated estimates of uncertainty (e.g. confidence intervals)
☐	☒	For null hypothesis testing, the test statistic (e.g. *F*, *t*, *r*) with confidence intervals, effect sizes, degrees of freedom and *P* value noted<br>*Give P values as exact values whenever suitable.*
☒	☐	For Bayesian analysis, information on the choice of priors and Markov chain Monte Carlo settings
☒	☐	For hierarchical and complex designs, identification of the appropriate level for tests and full reporting of outcomes
☒	☐	Estimates of effect sizes (e.g. Cohen's *d*, Pearson's *r*), indicating how they were calculated

Our web collection on statistics for biologists contains articles on many of the points above.

Software and code

Policy information about availability of computer code

Data collection	ForteBio-Data Acquisition 8.2 (for Octet)
Data analysis	ForteBio - Data Analysis 8.2 (for Octet)<br>GenePix Pro 7<br>GraphPad Prism 9.4.1 (458) Macintosh Version

For manuscripts utilizing custom algorithms or software that are central to the research but not yet described in published literature, software must be made available to editors and reviewers. We strongly encourage code deposition in a community repository (e.g. GitHub). See the Nature Portfolio guidelines for submitting code & software for further information.

Data

Policy information about availability of data

All manuscripts must include a data availability statement. This statement should provide the following information, where applicable:

- Accession codes, unique identifiers, or web links for publicly available datasets
- A description of any restrictions on data availability
- For clinical datasets or third party data, please ensure that the statement adheres to our policy

The data that support the findings from this study are available from corresponding authors upon reasonable request. We have additional manuscripts in development that may include or refer to some of the source data, so we are not ready to release all of the source data yet.

Research involving human participants, their data, or biological material

Policy information about studies with human participants or human data. See also policy information about sex, gender (identity/presentation), and sexual orientation and race, ethnicity and racism.

Reporting on sex and gender	No research performed on human subjects
Reporting on race, ethnicity, or other socially relevant groupings	Not applicable
Population characteristics	Not applicable
Recruitment	Not applicable
Ethics oversight	Not applicable

Note that full information on the approval of the study protocol must also be provided in the manuscript.

Field-specific reporting

Please select the one below that is the best fit for your research. If you are not sure, read the appropriate sections before making your selection.

[x] Life sciences [ ] Behavioural & social sciences [ ] Ecological, evolutionary & environmental sciences

For a reference copy of the document with all sections, see nature.com/documents/nr-reporting-summary-flat.pdf

Life sciences study design

All studies must disclose on these points even when the disclosure is negative.

Sample size	Group sample sizes of 5 in both groups achieve 83% power to detect a difference between the group proportions of 0.849. The proportion in the treatment group is assumed to be 0.001 under the null hypothesis and 0.85 under the alternative hypothesis. The proportion in the control group is 0.001. The test statistic used is the two-sided Fisher's Exact Test. The significance level of the test is targeted at 0.05. To control the overall Type I error rate, we propose a Dunnett's correction and a Tukey or Bonferroni adjustment depending on the a priori number of comparisons. To expedite all power calculations, control mortality is assumed to be 99% in all cases. In this study, each of 4 test groups (5-6 animals per group) will be compared against a control group of the same virus clade. The power to detect a difference in mortality is at least 85% when the vaccine group mortality is no more than 2.5%.
Data exclusions	No data excluded from analysis. Two high dose vaccine groups were largely excluded from description in the manuscript, but all study groups were analyzed together.
Replication	All assays included in the manuscript were run at least twice and all included successful technical replicates. Further details are included in the manuscript.
Randomization	Hartley guinea pigs were randomly allocated into study groups of 5 animals.
Blinding	Study animals were purchased and randomly assigned to experimental groups. Investigators were not blinded during data collection for this study. Study groups required administration of two doses of vaccine construct, so knowledge of groups with certain doses and constructs was required. Additionally, animal IDs used for daily welfare checks contained information on dosing and construct used.

Reporting for specific materials, systems and methods

We require information from authors about some types of materials, experimental systems and methods used in many studies. Here, indicate whether each material, system or method listed is relevant to your study. If you are not sure if a list item applies to your research, read the appropriate section before selecting a response.

Materials & experimental systems

n/a	Involved in the study	
[ ]	[x]	Antibodies
[ ]	[x]	Eukaryotic cell lines
[x]	[ ]	Palaeontology and archaeology
[ ]	[x]	Animals and other organisms
[x]	[ ]	Clinical data
[x]	[ ]	Dual use research of concern
[x]	[ ]	Plants

Methods

n/a	Involved in the study	
[x]	[ ]	ChIP-seq
[x]	[ ]	Flow cytometry
[x]	[ ]	MRI-based neuroimaging

Antibodies

Antibodies used	HuMAB 37.7H - GPC (Ab00377H - Zalgen), HuMab 3.3B - GP1(Ab00033B - Zalgen), HuMAb 22.5D - GP1(Ab00225D - Zelgen), and an LASV Nucleoprotein rabbit polyclonal antibody (GeneTex, Cat# GTX134883)
Validation	All antibodies were provided from Zalgen Labs. Validation using in-house ELISA is detailed in the manuscript. Other citations using Zalgen Lassa antibodies include:<br>- PMID: 26981786, 24651047, 27161536, 28869611, 19105844, 32994446, 28572385, 27531397<br>Antibody for immunohistochemistry staining - validated in-house: LASV nucleoprotein rabbit polyclonal antibody (GeneTex, Cat# GTX134883)

Eukaryotic cell lines

Policy information about cell lines and Sex and Gender in Research

Cell line source(s)	- ATCC: Vero-CCL-81 (Vero E6) - cell line was initiated in 1962 from the kidney tissue derived from a normal, adult female African green monkey.<br>- Modified Vero-CCL-81 (Vero NY) - given from a collaborator at UTMB - derived from cell line initiated in 1962 from the kidney tissue derived from a normal, adult female African green monkey. It is permissive to Lassa infection.
Authentication	- ATCC Vero-CCL-81: STR profiling authentication performed by ATCC.<br>- Vero NY cells not authenticated
Mycoplasma contamination	All negative for Mycoplasma
Commonly misidentified lines (See ICLAC register)	None

Animals and other research organisms

Policy information about studies involving animals; ARRIVE guidelines recommended for reporting animal research, and Sex and Gender in Research

Laboratory animals	Hartley guinea pigs - 4-6 weeks old.
Wild animals	None
Reporting on sex	All female animals used. As this study was conducted in high biocontainment (ABSL-4) there were complications with space and high risk during procedures. All female animals were also chosen to reduce the chance of behavioral issues (fighting) with co-housed animals.
Field-collected samples	Study did not include field-collected samples.
Ethics oversight	All study protocols approved by the University of Texas Medical Branch (UTMB) Institutional Animal Care and Use Committee (IACUC) and animal care handled by institutional Animal Resources Center staff.

Note that full information on the approval of the study protocol must also be provided in the manuscript.
