# Supplementary material for: A Lassa virus mRNA vaccine confers protection but does not require neutralizing antibody in a guinea pig model of infection
Source: Nat Commun. 2023 Sep 12;14:5603. doi: 10.1038/s41467-023-41376-6 (PMC10497546; doi:10.1038/s41467-023-41376-6)
Supplement: Supplementary file 3 — Source Data [file 41467_2023_41376_MOESM3_ESM.zip › Manuscript Source Data/Figure 7/Figure 7.pptx]

## Slide 1
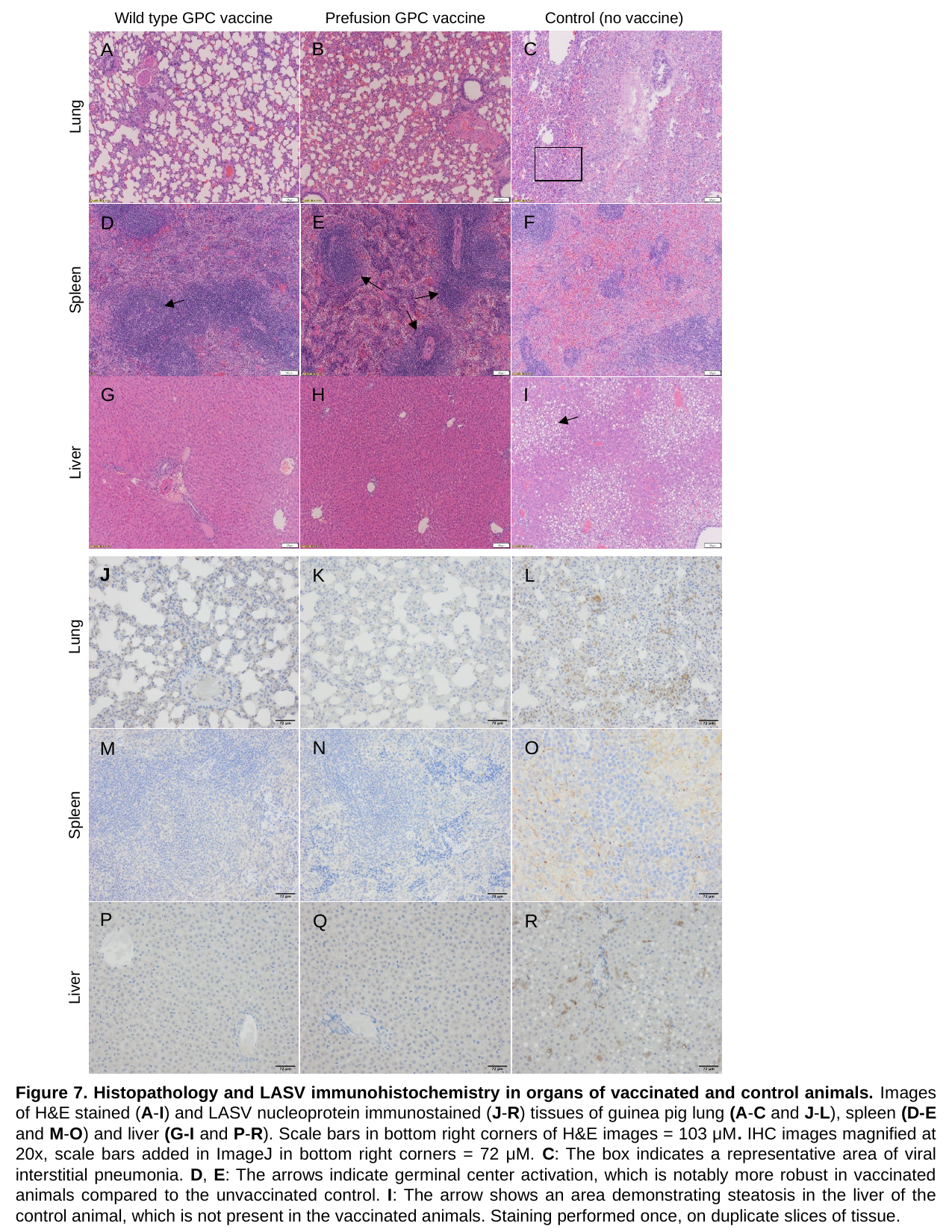

Prefusion GPC vaccine
Control (no vaccine)
Wild type GPC vaccine
B
C
A
Lung
E
F
D
Spleen
H
I
G
Liver
J
L
K
Lung
N
O
M
Spleen
P
Q
R
Liver
Figure 7. Histopathology and LASV immunohistochemistry in organs of vaccinated and control animals. Images of H&E stained (A-I) and LASV nucleoprotein immunostained (J-R) tissues of guinea pig lung (A-C and J-L), spleen (D-E and M-O) and liver (G-I and P-R). Scale bars in bottom right corners of H&E images = 103 μM. IHC images magnified at 20x, scale bars added in ImageJ in bottom right corners = 72 μM. C: The box indicates a representative area of viral interstitial pneumonia. D, E: The arrows indicate germinal center activation, which is notably more robust in vaccinated animals compared to the unvaccinated control. I: The arrow shows an area demonstrating steatosis in the liver of the control animal, which is not present in the vaccinated animals. Staining performed once, on duplicate slices of tissue.
