# Supplementary material for: A Lassa virus mRNA vaccine confers protection but does not require neutralizing antibody in a guinea pig model of infection
Source: Nat Commun. 2023 Sep 12;14:5603. doi: 10.1038/s41467-023-41376-6 (PMC10497546; doi:10.1038/s41467-023-41376-6)
Supplement: Supplementary file 3 — Source Data [file 41467_2023_41376_MOESM3_ESM.zip › Manuscript Source Data/Figure 6/Figure 6.pptx]

## Slide 1
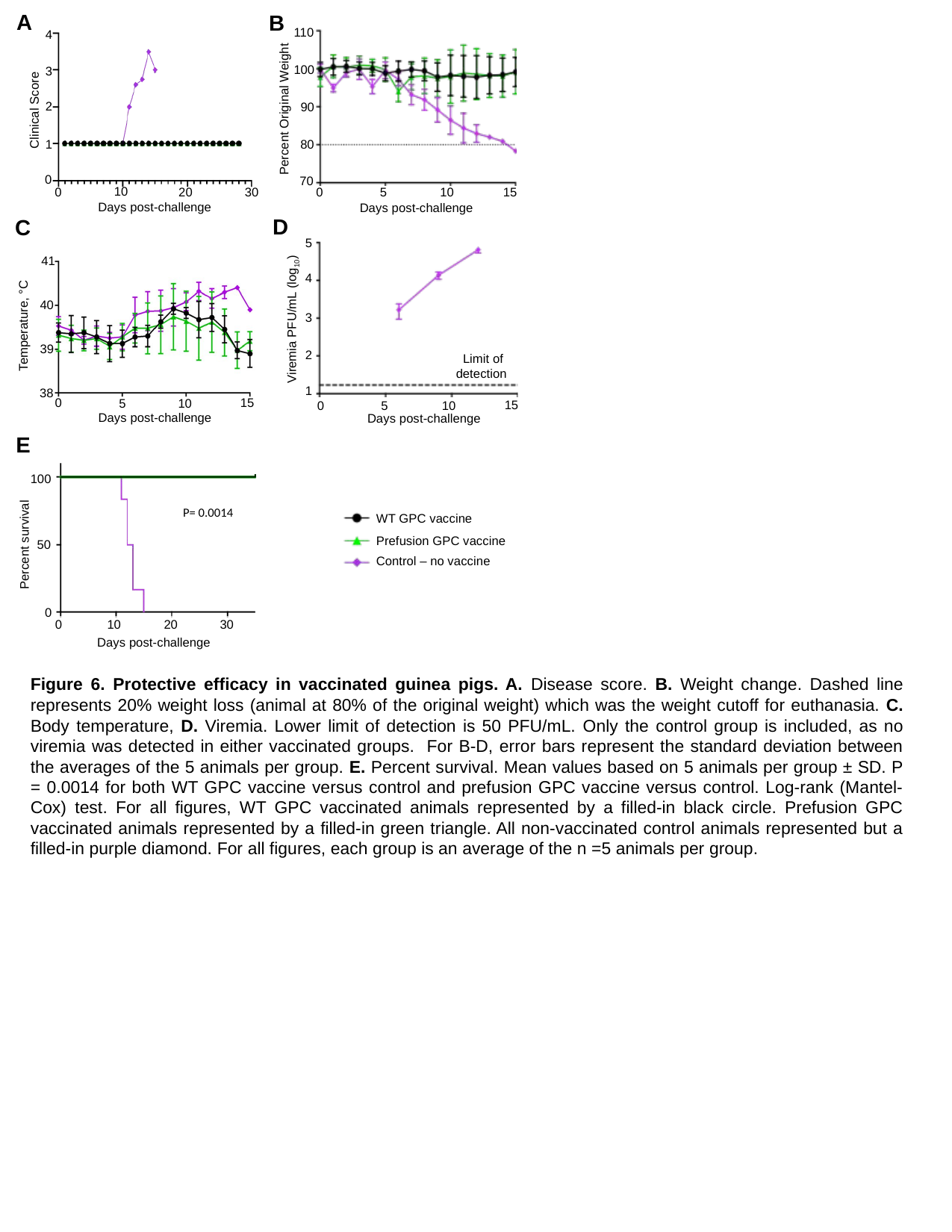

A
B
110
4
100
3
Clinical Score
Percent Original Weight
2
90
1
80
0
70
10
10
15
0
20
30
0
5
Days post-challenge
Days post-challenge
D
C
5
41
4
40
Viremia PFU/mL (log10)
3
Temperature, °C
39
2
Limit of
detection
1
38
0
15
5
10
15
10
5
0
Days post-challenge
Days post-challenge
E
100
P= 0.0014
WT GPC vaccine
Prefusion GPC vaccine
Percent survival
50
Control – no vaccine
0
0
10
20
30
Days post-challenge
Figure 6. Protective efficacy in vaccinated guinea pigs. A. Disease score. B. Weight change. Dashed line represents 20% weight loss (animal at 80% of the original weight) which was the weight cutoff for euthanasia. C. Body temperature, D. Viremia. Lower limit of detection is 50 PFU/mL. Only the control group is included, as no viremia was detected in either vaccinated groups. For B-D, error bars represent the standard deviation between the averages of the 5 animals per group. E. Percent survival. Mean values based on 5 animals per group ± SD. P = 0.0014 for both WT GPC vaccine versus control and prefusion GPC vaccine versus control. Log-rank (Mantel-Cox) test. For all figures, WT GPC vaccinated animals represented by a filled-in black circle. Prefusion GPC vaccinated animals represented by a filled-in green triangle. All non-vaccinated control animals represented but a filled-in purple diamond. For all figures, each group is an average of the n =5 animals per group.
