# Supplementary material for: A Lassa virus mRNA vaccine confers protection but does not require neutralizing antibody in a guinea pig model of infection
Source: Nat Commun. 2023 Sep 12;14:5603. doi: 10.1038/s41467-023-41376-6 (PMC10497546; doi:10.1038/s41467-023-41376-6)
Supplement: Supplementary file 3 — Source Data [file 41467_2023_41376_MOESM3_ESM.zip › Manuscript Source Data/Figure 1/Figure 1.pptx]

## Slide 1
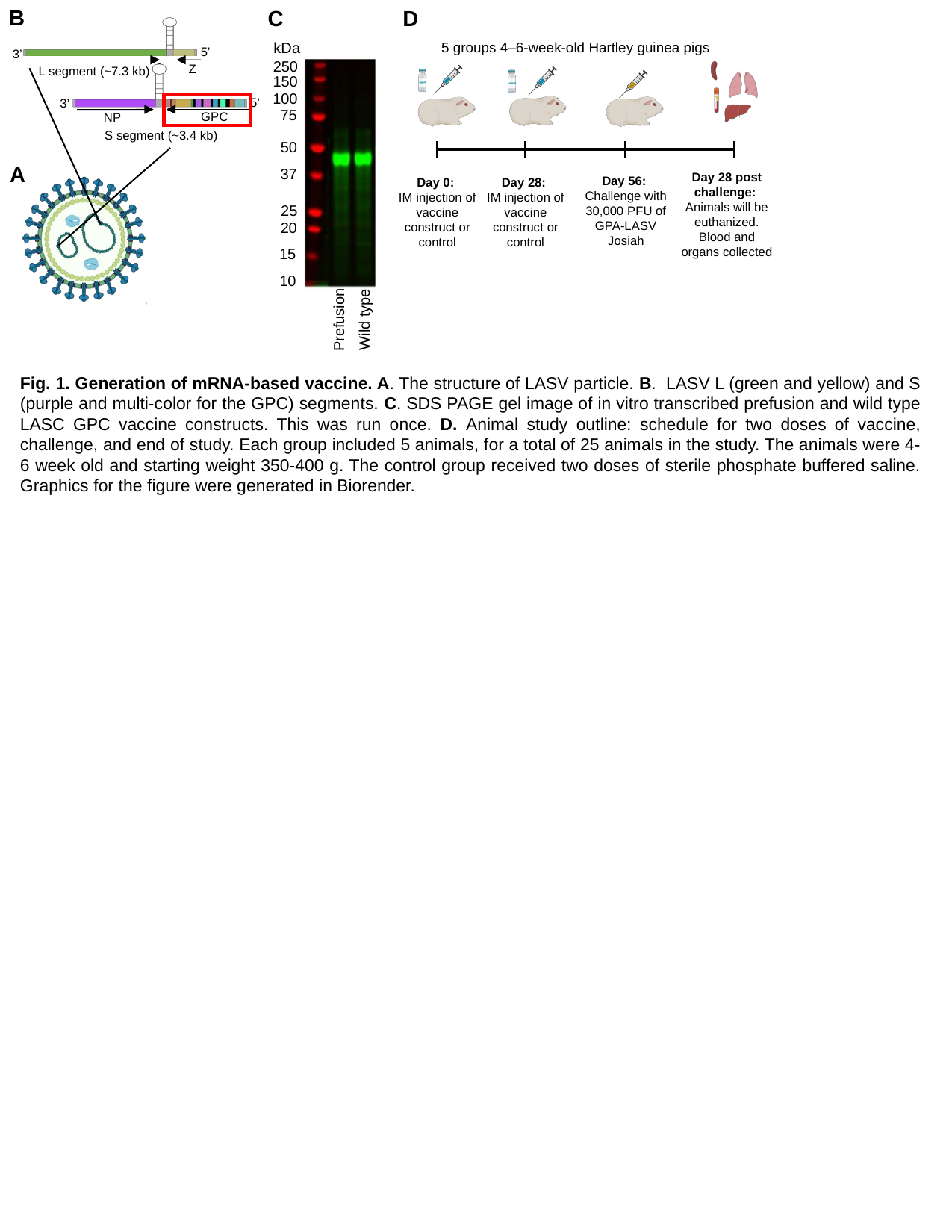

B
D
C
5’
3’
Z
L segment (~7.3 kb)
kDa
5 groups 4–6-week-old Hartley guinea pigs
250
5’
3’
GPC
NP
150
100
75
S segment (~3.4 kb)
50
A
37
Day 28 post challenge:
Animals will be euthanized. Blood and organs collected
Day 56:
Challenge with 30,000 PFU of GPA-LASV Josiah
Day 0:
IM injection of vaccine construct or control
Day 28:
IM injection of vaccine construct or control
25
20
15
10
Prefusion
Wild type
Fig. 1. Generation of mRNA-based vaccine. A. The structure of LASV particle. B. LASV L (green and yellow) and S (purple and multi-color for the GPC) segments. C. SDS PAGE gel image of in vitro transcribed prefusion and wild type LASC GPC vaccine constructs. This was run once. D. Animal study outline: schedule for two doses of vaccine, challenge, and end of study. Each group included 5 animals, for a total of 25 animals in the study. The animals were 4-6 week old and starting weight 350-400 g. The control group received two doses of sterile phosphate buffered saline. Graphics for the figure were generated in Biorender.
