# Supplementary material for: A Lassa virus mRNA vaccine confers protection but does not require neutralizing antibody in a guinea pig model of infection
Source: Nat Commun. 2023 Sep 12;14:5603. doi: 10.1038/s41467-023-41376-6 (PMC10497546; doi:10.1038/s41467-023-41376-6)
Supplement: Supplementary file 3 — Source Data [file 41467_2023_41376_MOESM3_ESM.zip › Manuscript Source Data/Figure 4/Figure 4.pptx]

## Slide 1
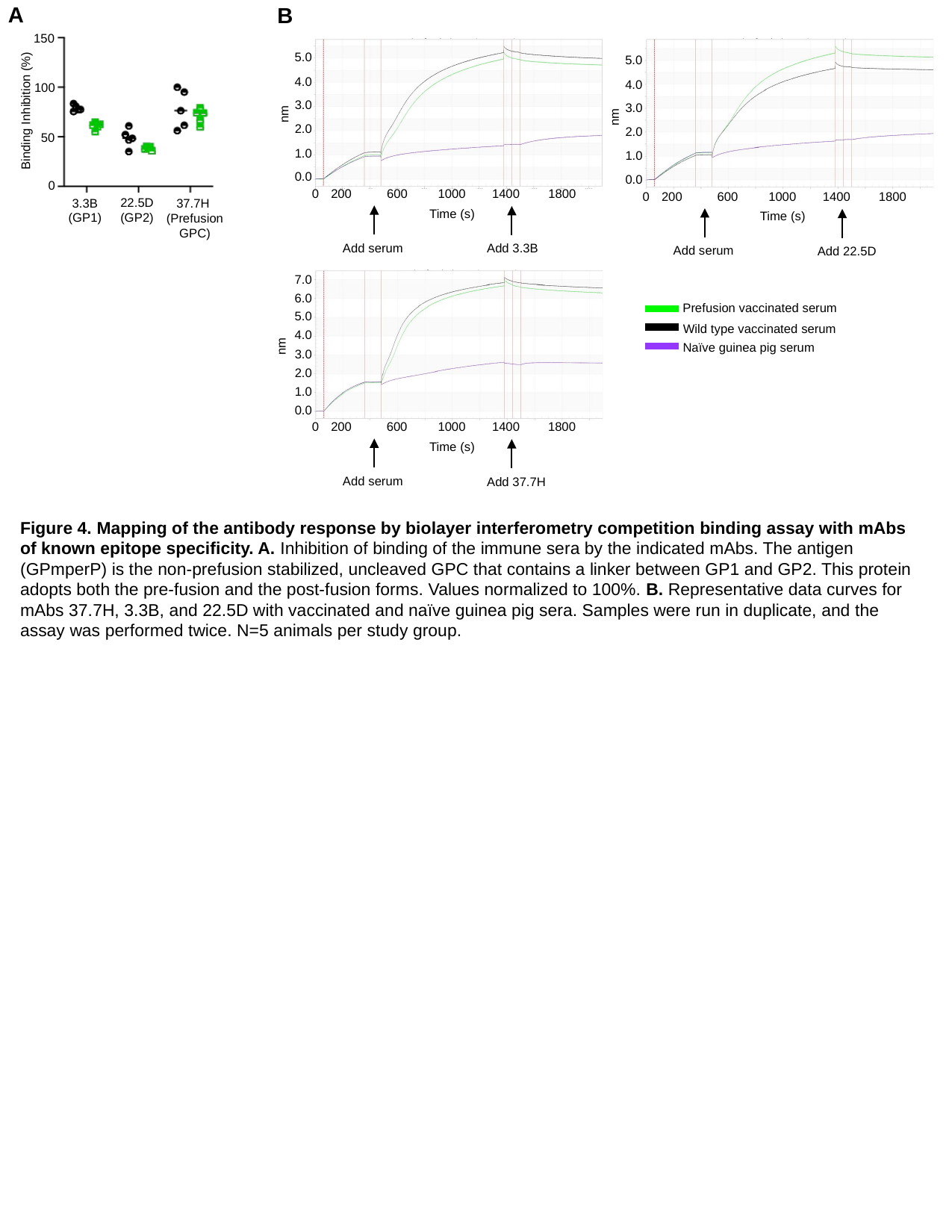

A
B
150
5.0
5.0
4.0
4.0
100
3.0
3.0
Binding Inhibition (%)
nm
nm
2.0
2.0
50
1.0
1.0
0.0
0.0
0
1000
1400
1800
600
0
200
1000
1400
1800
600
0
200
22.5D (GP2)
3.3B
(GP1)
37.7H
(Prefusion GPC)
Time (s)
Time (s)
Add serum
Add 3.3B
Add serum
Add 22.5D
7.0
6.0
Prefusion vaccinated serum
5.0
Wild type vaccinated serum
4.0
nm
Naïve guinea pig serum
3.0
2.0
1.0
0.0
1000
1400
1800
600
0
200
Time (s)
Add serum
Add 37.7H
Figure 4. Mapping of the antibody response by biolayer interferometry competition binding assay with mAbs of known epitope specificity. A. Inhibition of binding of the immune sera by the indicated mAbs. The antigen (GPmperP) is the non-prefusion stabilized, uncleaved GPC that contains a linker between GP1 and GP2. This protein adopts both the pre-fusion and the post-fusion forms. Values normalized to 100%. B. Representative data curves for mAbs 37.7H, 3.3B, and 22.5D with vaccinated and naïve guinea pig sera. Samples were run in duplicate, and the assay was performed twice. N=5 animals per study group.
