# Supplementary material for: A Lassa virus mRNA vaccine confers protection but does not require neutralizing antibody in a guinea pig model of infection
Source: Nat Commun. 2023 Sep 12;14:5603. doi: 10.1038/s41467-023-41376-6 (PMC10497546; doi:10.1038/s41467-023-41376-6)
Supplement: Supplementary file 3 — Source Data [file 41467_2023_41376_MOESM3_ESM.zip › Manuscript Source Data/Figure 3/Figure 3.pptx]

## Slide 1
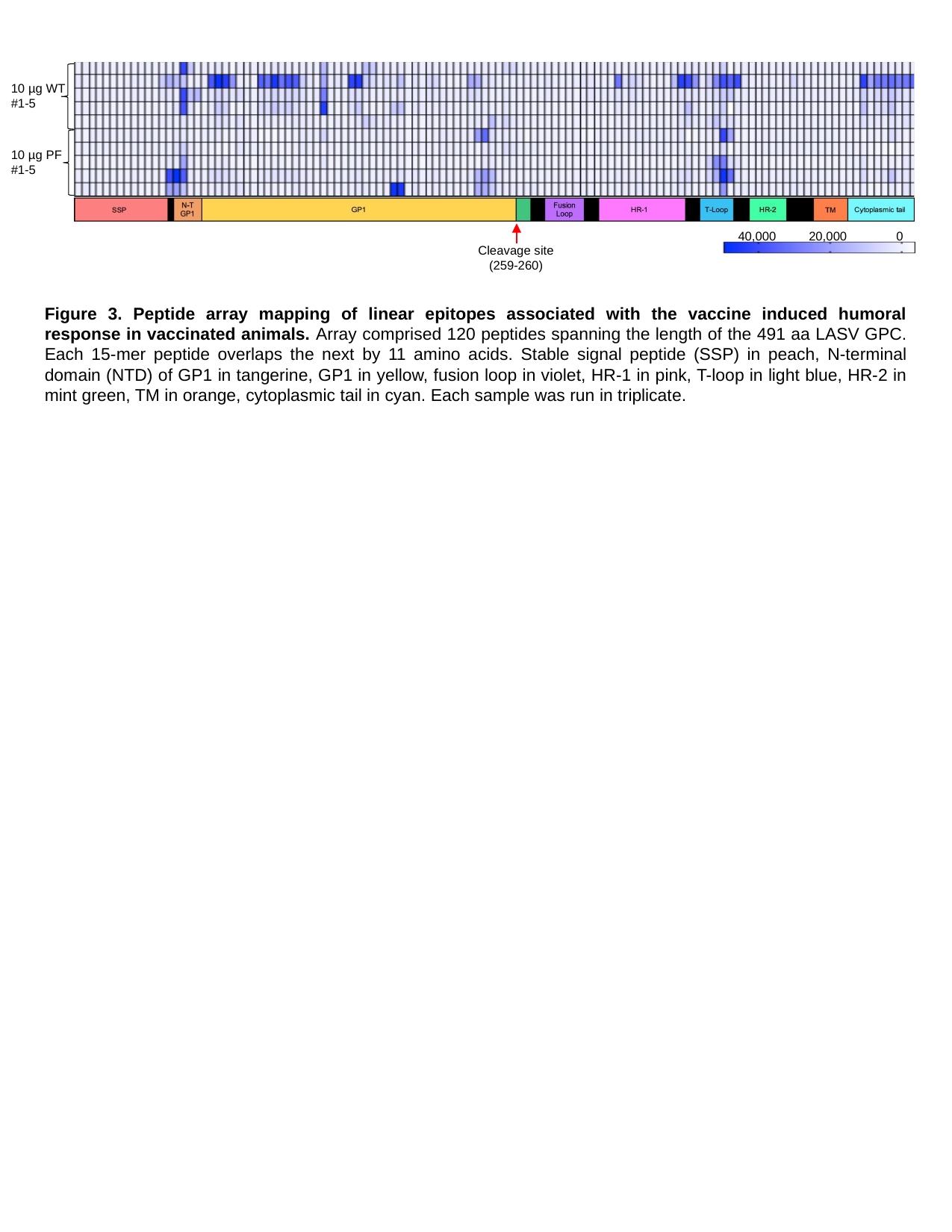

10 µg WT #1-5
10 µg PF #1-5
40,000
 0
20,000
Cleavage site (259-260)
Figure 3. Peptide array mapping of linear epitopes associated with the vaccine induced humoral response in vaccinated animals. Array comprised 120 peptides spanning the length of the 491 aa LASV GPC. Each 15-mer peptide overlaps the next by 11 amino acids. Stable signal peptide (SSP) in peach, N-terminal domain (NTD) of GP1 in tangerine, GP1 in yellow, fusion loop in violet, HR-1 in pink, T-loop in light blue, HR-2 in mint green, TM in orange, cytoplasmic tail in cyan. Each sample was run in triplicate.
