# Supplementary material for: A Lassa virus mRNA vaccine confers protection but does not require neutralizing antibody in a guinea pig model of infection
Source: Nat Commun. 2023 Sep 12;14:5603. doi: 10.1038/s41467-023-41376-6 (PMC10497546; doi:10.1038/s41467-023-41376-6)
Supplement: Supplementary file 3 — Source Data [file 41467_2023_41376_MOESM3_ESM.zip › Manuscript Source Data/Figure 2/Figure 2.pptx]

## Slide 1
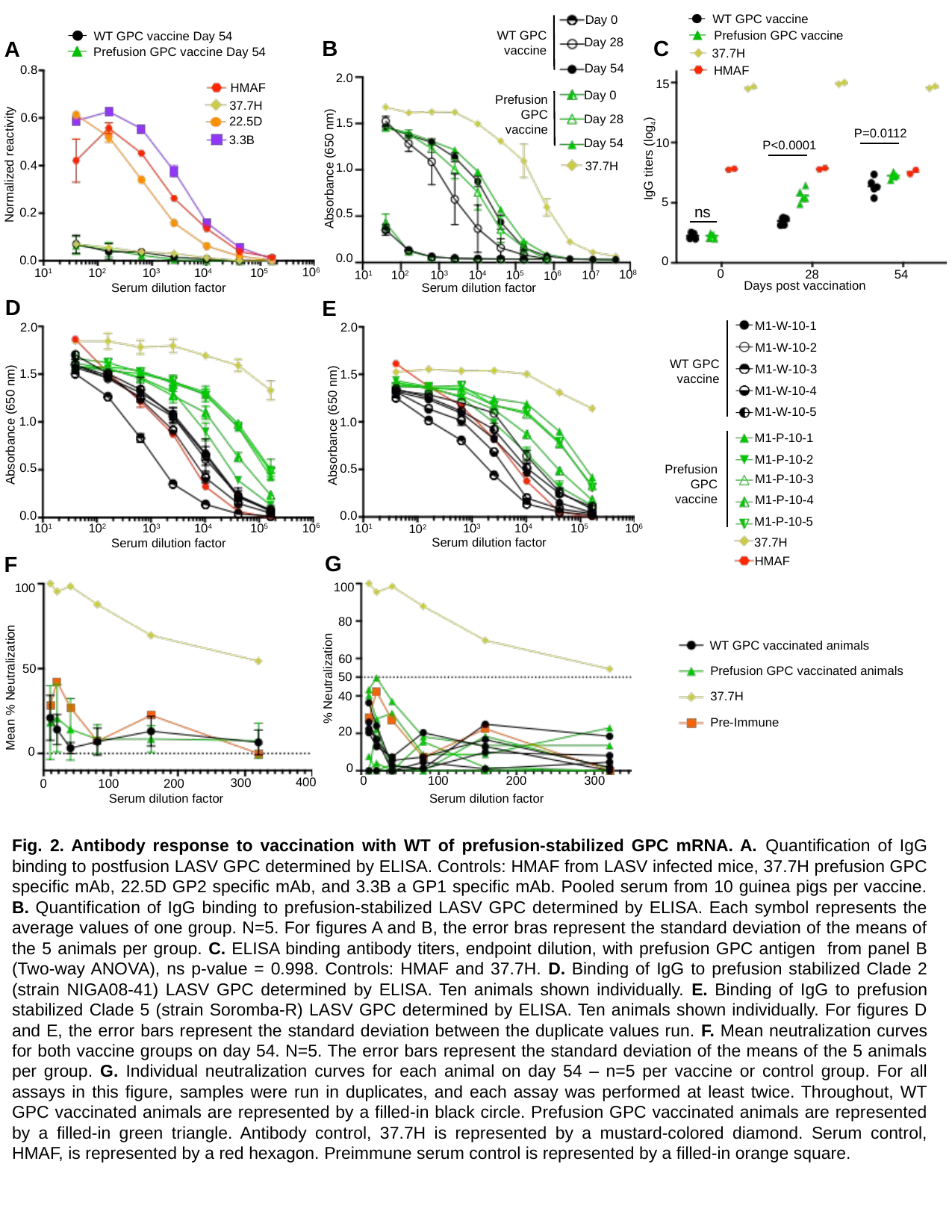

WT GPC vaccine
Day 0
Prefusion GPC vaccine
WT GPC vaccine
WT GPC vaccine Day 54
Day 28
C
B
A
Prefusion GPC vaccine Day 54
37.7H
Day 54
0.8
HMAF
2.0
15
HMAF
Day 0
Prefusion GPC vaccine
37.7H
0.6
Day 28
22.5D
1.5
P=0.0112
3.3B
Day 54
10
P<0.0001
IgG titers (log4)
Normalized reactivity
0.4
Absorbance (650 nm)
37.7H
1.0
5
ns
0.2
0.5
0.0
0.0
0
106
105
104
103
101
102
0
108
28
54
107
101
102
103
104
105
106
Days post vaccination
Serum dilution factor
Serum dilution factor
D
E
M1-W-10-1
2.0
2.0
M1-W-10-2
WT GPC vaccine
M1-W-10-3
1.5
1.5
M1-W-10-4
M1-W-10-5
1.0
Absorbance (650 nm)
1.0
Absorbance (650 nm)
M1-P-10-1
M1-P-10-2
0.5
0.5
Prefusion GPC vaccine
M1-P-10-3
M1-P-10-4
0.0
0.0
M1-P-10-5
106
106
105
104
103
101
102
105
104
103
101
102
Serum dilution factor
37.7H
Serum dilution factor
G
F
HMAF
100
100
80
WT GPC vaccinated animals
60
50
Prefusion GPC vaccinated animals
% Neutralization
50
Mean % Neutralization
40
37.7H
Pre-Immune
20
0
0
300
0
100
200
400
300
0
100
200
Serum dilution factor
Serum dilution factor
Fig. 2. Antibody response to vaccination with WT of prefusion-stabilized GPC mRNA. A. Quantification of IgG binding to postfusion LASV GPC determined by ELISA. Controls: HMAF from LASV infected mice, 37.7H prefusion GPC specific mAb, 22.5D GP2 specific mAb, and 3.3B a GP1 specific mAb. Pooled serum from 10 guinea pigs per vaccine. B. Quantification of IgG binding to prefusion-stabilized LASV GPC determined by ELISA. Each symbol represents the average values of one group. N=5. For figures A and B, the error bras represent the standard deviation of the means of the 5 animals per group. C. ELISA binding antibody titers, endpoint dilution, with prefusion GPC antigen from panel B (Two-way ANOVA), ns p-value = 0.998. Controls: HMAF and 37.7H. D. Binding of IgG to prefusion stabilized Clade 2 (strain NIGA08-41) LASV GPC determined by ELISA. Ten animals shown individually. E. Binding of IgG to prefusion stabilized Clade 5 (strain Soromba-R) LASV GPC determined by ELISA. Ten animals shown individually. For figures D and E, the error bars represent the standard deviation between the duplicate values run. F. Mean neutralization curves for both vaccine groups on day 54. N=5. The error bars represent the standard deviation of the means of the 5 animals per group. G. Individual neutralization curves for each animal on day 54 – n=5 per vaccine or control group. For all assays in this figure, samples were run in duplicates, and each assay was performed at least twice. Throughout, WT GPC vaccinated animals are represented by a filled-in black circle. Prefusion GPC vaccinated animals are represented by a filled-in green triangle. Antibody control, 37.7H is represented by a mustard-colored diamond. Serum control, HMAF, is represented by a red hexagon. Preimmune serum control is represented by a filled-in orange square.
