# Supplementary material for: A Lassa virus mRNA vaccine confers protection but does not require neutralizing antibody in a guinea pig model of infection
Source: Nat Commun. 2023 Sep 12;14:5603. doi: 10.1038/s41467-023-41376-6 (PMC10497546; doi:10.1038/s41467-023-41376-6)
Supplement: Supplementary file 3 — Source Data [file 41467_2023_41376_MOESM3_ESM.zip › Manuscript Source Data/Figure 5/Figure 5.pptx]

## Slide 1
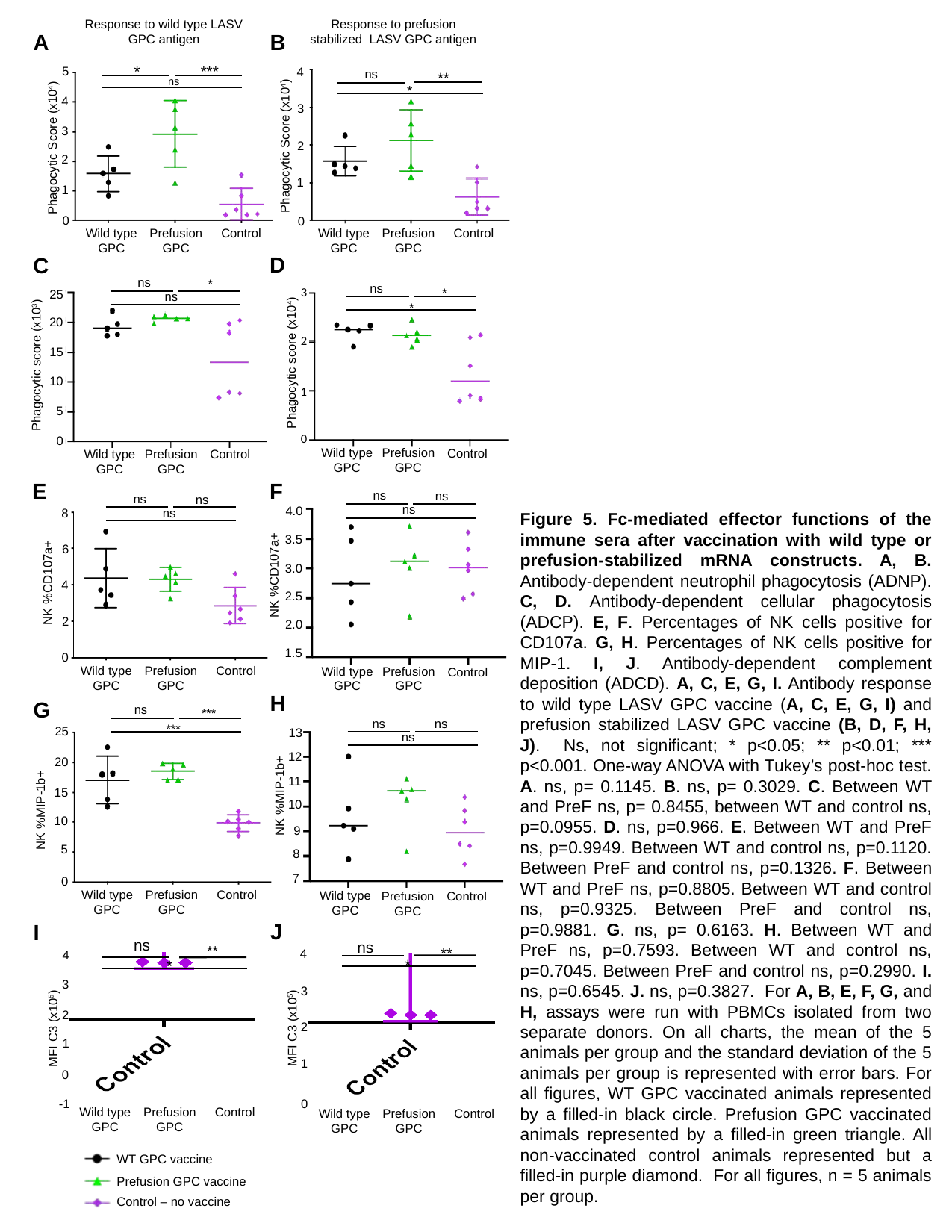

Response to wild type LASV GPC antigen
Response to prefusion stabilized LASV GPC antigen
A
B
***
*
5
4
ns
**
ns
*
4
3
3
2
Phagocytic Score (x104)
Phagocytic Score (x104)
2
1
1
0
0
Wild type
GPC
Control
Prefusion
GPC
Wild type
GPC
Control
Prefusion
GPC
D
C
ns
*
ns
3
*
25
ns
*
20
2
15
Phagocytic score (x103)
Phagocytic score (x104)
10
1
5
0
0
Wild type
GPC
Prefusion
GPC
Control
Wild type
GPC
Control
Prefusion
GPC
F
E
ns
ns
ns
ns
ns
4.0
ns
8
3.5
6
3.0
NK %CD107a+
NK %CD107a+
4
2.5
2
2.0
1.5
0
Wild type
GPC
Control
Prefusion
GPC
Wild type
GPC
Prefusion
GPC
Control
H
G
ns
***
ns
ns
***
25
13
ns
12
20
11
15
NK %MIP-1b+
10
NK %MIP-1b+
10
9
5
8
7
0
Wild type
GPC
Control
Prefusion
GPC
Wild type
GPC
Control
Prefusion
GPC
J
I
ns
ns
**
**
4
4
*
*
3
3
2
2
MFI C3 (x105)
MFI C3 (x105)
1
1
0
-1
0
Wild type
GPC
Control
Prefusion
GPC
Wild type
GPC
Control
Prefusion
GPC
WT GPC vaccine
Prefusion GPC vaccine
Control – no vaccine
